# Current Insights into miRNA and lncRNA Dysregulation in Diabetes: Signal Transduction, Clinical Trials and Biomarker Discovery

**DOI:** 10.3390/ph15101269

**Published:** 2022-10-14

**Authors:** Amitkumar Pandey, Saiprasad Ajgaonkar, Nikita Jadhav, Praful Saha, Pranay Gurav, Sangita Panda, Dilip Mehta, Sujit Nair

**Affiliations:** 1 Viridis Biopharma Pvt. Ltd., Mumbai 400 022, India; 2 Synergia Life Sciences Pvt. Ltd., Mumbai 400 022, India

**Keywords:** microRNA, lncRNA, biological network, signal transduction, clinical trial, epigenetics, diabetes, diabetic nephropathy, noncoding RNA, diabetic neuropathy, miRNA, diabetic retinopathy, biomarker

## Abstract

Diabetes is one of the most frequently occurring metabolic disorders, affecting almost one tenth of the global population. Despite advances in antihyperglycemic therapeutics, the management of diabetes is limited due to its complexity and associated comorbidities, including diabetic neuropathy, diabetic nephropathy and diabetic retinopathy. Noncoding RNAs (ncRNAs), including microRNAs (miRNAs) and long noncoding RNAs (lncRNAs), are involved in the regulation of gene expression as well as various disease pathways in humans. Several ncRNAs are dysregulated in diabetes and are responsible for modulating the expression of various genes that contribute to the ‘symptom complex’ in diabetes. We review various miRNAs and lncRNAs implicated in diabetes and delineate ncRNA biological networks as well as key ncRNA targets in diabetes. Further, we discuss the spatial regulation of ncRNAs and their role(s) as prognostic markers in diabetes. We also shed light on the molecular mechanisms of signal transduction with diabetes-associated ncRNAs and ncRNA-mediated epigenetic events. Lastly, we summarize clinical trials on diabetes-associated ncRNAs and discuss the functional relevance of the dysregulated ncRNA interactome in diabetes. This knowledge will facilitate the identification of putative biomarkers for the therapeutic management of diabetes and its comorbidities. Taken together, the elucidation of the architecture of signature ncRNA regulatory networks in diabetes may enable the identification of novel biomarkers in the discovery pipeline for diabetes, which may lead to better management of this metabolic disorder.

## 1. Introduction

Diabetes is the most commonly occurring metabolic disorder, affecting almost 366 million people, and is estimated to affect 10% of the world population by 2035 [1]. It is characterized by elevated blood glucose (hyperglycemia) occurring due to malfunctions in insulin action, insulin production or both [2]. There are three main types of diabetes mellitus—type 1 diabetes (T1D), type 2 diabetes (T2D) and gestational diabetes mellitus (GDM). Type 1 is caused by an autoimmune reaction destroying beta cells, which eventually leads to hampered production of insulin in the body. T2D occurs when the body fails to use insulin properly, causing dysregulation of blood glucose. Gestational diabetes is a condition that develops during pregnancy. However, gestational diabetes usually subsides post-pregnancy [3]. Diabetes was the seventh leading cause of death in the U.S. in the year 2017 [4]. Globally, in the year 2019, the prevalence of diabetes was estimated to be 9.3% (463 million worldwide) and was predicted to rise to 10.2% (578 million globally) by the year 2030 and to 10.9% (700 million globally) by 2040 [5].

Noncoding RNAs (ncRNAs) are a heterogenous group of RNA molecules which do not code for any proteins but play a major role in post-transcriptional gene regulation and epigenetic gene silencing. Introns which are present in both protein-coding and non-protein-coding genes are the major source of regulatory noncoding RNAs [6]. Examples of ncRNAs include microRNAs (miRNAs), long noncoding RNAs (lncRNAs), small interfering RNAs (siRNAs), circular RNAs (circRNAs), competing endogenous RNAs (ceRNAs) and piwi-interacting RNAs (piRNAs) [7]. MiRNAs are a class of small endogenous molecules comprising around 21 nucleotides. They are known to regulate cell cycle proliferation, differentiation, angiogenesis and apoptosis by repressing biomolecules such as RNA transcripts, which regulate these processes, or via the degradation of mRNA [8]. Studies show [9] that miRNAs have complementarity to their target genes and, thus, can regulate different mRNA targets. miRNAs are produced from a double-stranded stem–loop structure generated from an endogenous single-stranded precursor, which is further processed by two endonucleases, RNase III enzyme drosha and RNase III endonuclease dicer complex. It has been reported that the knockdown of the dicer enzyme-encoding gene in pancreatic beta cells leads to the development of diabetes [10]. MiRNA expression studies have shown that miR-375 is highly expressed in islet cells. The dysregulation of miR-375 affects the insulin-secreting beta cells. Other abundantly expressed miRNAs in islet cells, such as miR-29a, miR-29b, miR-200 and miR-7, influence the synthesis of insulin by playing a role in the development and proliferation of beta cells and, thus, can be proposed as biomarkers for diabetes [11].

Biological networks have been useful in studies to decipher disease etiologies and biological mechanisms, and in studies predicting therapeutic responses at system as well as molecular levels [12]. Biological networks help to find and prioritize candidate genes responsible for various diseases, facilitate target identification and are involved in drug discovery by capturing therapeutic responses and identifying systemic perturbations and disease-associated subnetworks [13]. We have previously elucidated the ncRNA interactome in cancer chemoprevention [7,14,15], mesothelioma [8], prostate cancer [16] and neuropathic pain [17], and also delineated miRNA-lncRNA interactions [18]. Cell-free RNAs, also called extracellular RNAs [19], are released into the circulatory system as a result of cell necrosis or apoptosis [20,21]. Cell-free ncRNAs have been observed to be dysregulated in peripheral blood samples of T2D patients. For example, miR-29a and miR-29b were upregulated, whereas lncRNA H19 was downregulated [22], exosomal miR-20b-5p was upregulated [23] and miR-210 was upregulated in peripheral blood [24]. Further, circRNA hsa-circCAMSAP1 in the peripheral blood of patients had a negative association with T2D [25], and hsa-circRNA11783-2 was reported to be downregulated in T2D [26]. 

Diabetic neuropathy is the most common symptom associated with diabetes, wherein the structure and function of the peripheral nerves are affected. We have recently discussed the molecular pathways underlying the health-beneficial role of K2-7 in diabetes and other complications, and also summarized several clinical trials involving K2-7 supplementation as a useful intervention [27]. In addition, we have reviewed the utility of K2-7 supplementation in the global diet and underscored its beneficial effects in managing peripheral neuropathy resulting from diabetes [28]. Other conventional medications in the management of diabetes may cause unpleasant side effects to patients, eventually leading to non-compliance and even failure of treatment [29]. We have previously discussed the applicability of personalized medicine and emerging roles for clinical pharmacometrics and pharmacogenomics in cancer [30,31,32], which can be helpful in developing similar strategies for the management of diabetes and related complications. Despite advances in diabetes management, there is a need for newer therapeutic strategies to achieve better glycemic control. Hence, the identification of new biomarkers will enable the development of newer therapeutics and/or nutraceuticals for controlling diabetes and its complications. We review the key miRNAs and lncRNAs involved in diabetes, as well as their targets, by means of miRNA–miRNA, lncRNA–lncRNA, miRNA–target and lncRNA–target networks. The elucidation of the noncoding RNA interactome in diabetes will enable the identification of novel biomarkers and augment the development of newer therapeutic strategies to achieve better glycemic control.

## 2. Dysregulated Human ncRNAs in Diabetes

### 2.1. Dysregulated ncRNAs in T1D

#### 2.1.1. Upregulated miRNAs in T1D

A study [33] investigating the miRNA expression profile using plasma samples of 33 T1D patients reported that several miRNAs such as miR-21-5p, miR-101-3p, miR-103a-3p, miR-148b-3p, miR-155-5p, miR-200a-3p, miR-210-3p and miR-1275 were significantly upregulated. Among these miRNAs, the expressions of miR-21 and miR-101 were induced by inflammatory cytokines (TNF and IL-1β) [34], showing their role in beta-cell destruction mediated by cytokines. Further, miR-200a-3p regulates *TP53* in the apoptosis pathway along with other genes involved in the insulin pathway and innate immune system; miR-210-3p regulates *PTPN1* (tyrosine-protein phosphatase non-receptor type 1) in the insulin pathway; and *IRS2* (Insulin receptor substrate 2) is regulated by both miR-200a-3p and miR-103a-3p [33]. Interestingly, there was no difference detected in the miRNA expression between the control group and T1D patients diagnosed for more than 5 years. Barutta et al. [35] conducted a cross-sectional nested case–control study to examine the differential expression of miRNAs in the serum samples of type 1 diabetic patients. It was reported that miR-140-3p, miR-574-3p, miR-139-5p, miR-106a, miR-17, miR-486-3p, miR-16, miR-222 and miR-885-5p were significantly upregulated. CXCL10 is targeted by miR-16, resulting in the inhibition of pancreatic β-cells [36]. Vascular homeostasis is regulated by miR-222 by negatively regulating STAT5A (signal transducer and activator of transcription 5A) in T1D patients, showing its anti-angiogenic properties [37]. In a study [38] using isolated human islets exposed to high glucose concentrations, and the serum samples of 22 patients with T1D, miR-23a and miR-98 were reported to be highly upregulated and targeted important signaling pathways involved in β-cell regulation and islet homeostasis, indicating that they might be useful biomarkers for T1D.

miR-197 was seen to be highly overexpressed in the serum samples of children with new-onset T1D [39]. It was also suggested that residual beta-cell function can be efficiently predicted by miR-197. The study further reported a significant partial correlation between miR-197 and stimulated C-peptide levels, indicating the critical role of miR-197 in insulin production. Moreover, using human activated CD4+ T cells, Scherm et al. [40] reported that miR-142-3p played a crucial role in the inhibition of T-cell activation, causing impaired stability of Treg cells. Elevated levels of miR-142-3p were reported to be linked to epigenetic remodeling in Treg cells, leading to islet autoimmunity, which is an indication of the onset of T1D.

#### 2.1.2. Upregulated lncRNAs in T1D

lncRNA SRA was reported [41] to be overexpressed in the plasma samples of 25 patients with T1D as well as in CD4+ regulatory T cells (Tregs) induced with high glucose in the CD4+ MOLT4 human T lymphoblast cell line. lncRNA SRA modulates the expression of miR-146b, which is downstream of interleukin-1 receptor-associated kinase 1 (IRAK1)/protein kinase B (AKT)/S6 Kinase 1 (S6K1) signaling. However, lncRNA SRA and miR-146b reciprocally regulate each other, which indicates that lncRNA participates in crosstalk with IRAK1/AKT/mTOR signaling by competing with miR-146b. A positive association between lactate levels and hemoglobin A1c (HbA1c) was observed in the plasma of T1D patients. lncRNA SRA played a major role in the pathogenesis of T1D by inhibiting miR-146b via promotion of the IRAK1/lactate dehydrogenase A (LDHA)/phosphorylated LDHA (pLDHA) signaling pathway.

#### 2.1.3. Downregulated miRNAs in T1D

Significant downregulation of miR-155, miR-92a and miR-126 was seen in the serum samples of T1D patients [35]. miR-92a regulates NF-κB and other inflammatory signaling pathways, aiding in the progression of cardiovascular disease associated with diabetes [42]. Further, miR-126 plays a role in maintaining endothelial homeostasis, which is a common characteristic in diabetes [43]. In addition, miR-126 controls endothelial inflammation in patients with micro/macrovascular disorders associated with diabetes, which provides a link between diabetes-associated complications and lower levels of miR-126 [44].

### 2.2. Dysregulated ncRNAs in T2D

#### 2.2.1. Upregulated miRNAs in T2D

miR-29a was upregulated in the serum samples of type 2 diabetic patients [45]. It was concluded that the overexpression of miR-29a represses glucose uptake via insulin stimulation, eventually causing resistance to insulin. In another study investigating the dysregulation of miR-34a, using the plasma samples of patients with T2D [46], it was observed that miR-34a was significantly upregulated, which was associated with senescence in pancreatic β-cells with a decrease in SIRT1 activity [47]. Interestingly, circulating miR-1 and miR-133a showed a positive association with myocardial steatosis in a study including T2D patients [48]. The elevated expression of miR-1 and hsa-miR-133a in the serum samples of T2D patients was an indicator of myocardial steatosis. Amr et al. reported that the plasma level of miR-210 was critically elevated in T2D patients with coronary artery disease. Indeed, miR-210 is linked with the hypoxia pathway and upregulated in response to hypoxia-inducible factor HIF1α [49].

#### 2.2.2. Upregulated lncRNAs in T2D

LncRNA-p3134 was highly upregulated in a study [50] conducted using whole blood samples of T2D patients. Using bioinformatic analysis, it was reported that lncRNA-p3134 targets mRNAs, viz., TNS1, ASZ1, DIAPH1, IFNA14, ZNF436, MTMR3, CDK1, PPARD, TCF7, HUWE1 and CCND2 and regulates their biological functions. Sathishkumar et al. [51] studied the differential expression of lncRNAs in the peripheral-blood mononuclear cells (PBMCs) from patients with T2D wherein the lncRNAs ANRIL, ENST00000550337, GAS5, HOTAIR, lincRNA-p21 and PLUTO were significantly overexpressed in diabetic patients. The lncRNAs ANRIL, GAS5 and HOTAIR show a positive correlation with senescence-related biomarkers (p16, p21, p53 and β-galactosidase) and pro-inflammatory biomarkers (IL1- β, MCP1, IL6 and TNF-α) [51].

#### 2.2.3. Downregulated miRNAs in T2D

In a study comprising of 327 T2D patients with varying levels of urinary albumin-to-creatinine ratios (UACR) [52], it was observed that miR-130b was highly suppressed in the serum samples of the patient group with the highest UACR. It was also observed that serum miR-130b was negatively correlated with HbA_1c_ and HIF1-α levels. Additionally, the circulating serum miR-130b was closely related to blood glucose levels and insulin resistance inT2DM. The study further reported that miR-130b can be a biomarker for the early identification of diabetic nephropathy in T2D. Significant downregulation of let-7b was seen in the peripheral whole-blood samples of T2D patients [53]. miRNA let-7b targeted T2D-susceptibility genes such as *IGF2BP2*, *TSPAN8*, *CDKN2A* and *KCNQ1*. Additionally, downregulation of miR-214-3p and miR-27-3p was reported in the whole-blood samples of patients with T2D and also in a population with T2D risk factors [54], suggesting that they might be potential biomarkers for T2D.

#### 2.2.4. Downregulated lncRNAs in T2D

A study [55] was conducted in 200 participants divided into two groups (diabetic and healthy volunteers). The differential expression of lncRNA VIM-AS1 and CTBP1-AS2 was studied in the PBMCs of these participants, and significant downregulation of both these lncRNAs was reported. Further, lncRNA CTBP1-AS2 was positively correlated with low-density-lipoprotein cholesterol (LDL-C) levels and negatively correlated with high-density-lipoprotein cholesterol (HDL-C) levels. No significant correlation between metabolic features and lncRNA VIM-AS1 was observed in the study. The target genes of the lncRNAs CTBP1-AS2 and VIM-AS1 were significantly enriched in pathways relevant to glucose homeostasis such as the mTOR, Hippo, TGF-β, insulin and Wnt signaling pathways.

### 2.3. Dysregulated ncRNAs in Diabetic Retinopathy

#### 2.3.1. Upregulated miRNAs in Diabetic Retinopathy

Li et al. [56] explored novel non-proliferative diabetic retinopathy (NPDR)-specific circulating miRNAs in T2D mellitus patients. It was observed that miR-190a was highly expressed in the serum samples of NPDR patients. Further, it was noted [57] that miR-2116-5p and miR-3197 were critically upregulated in the serum samples of diabetic patients with retinopathy, which made it an important factor in differentiating between patients with and without DR. Notch homolog 2 (NOTCH2) was reported to be a target gene of miR-2116-5p in human embryonic kidney cells (HEK293T). Enhanced miR-20a-5p and -223-3p expression was seen in the serum samples of T2D patients with retinopathy, suggesting their critical role in the early detection of diabetic retinopathy [58].

#### 2.3.2. Upregulated lncRNAs in Diabetic Retinopathy

A study [59] using the serum samples of 80 diabetic patients with either non-diabetic retinopathy (NDR), NPDR or proliferative diabetic retinopathy (PDR) was conducted to investigate the differential expression of lncRNAs. lncRNA HOTAIR and lncRNA MALAT1 (metastasis-associated lung adenocarcinoma transcript 1) were reported to be significantly upregulated in all groups. MALAT1 is a highly conserved lncRNA playing an important role in diabetes progression by modulating genes at the transcription level. MALAT1 has also been reported to be a regulator of the hyperglycemia-induced inflammatory process [60]. HOX transcript antisense intergenic RNA (HOTAIR), located on chr.12q13.13, is expressed abnormally in several disorders involving T1D. It is involved in distinguishing between diabetic and non-diabetic retinopathy as the expression levels of HOTAIR are increased in diabetic retinopathy [59]. The lncRNA HOTAIR also plays a role in regulating glucose levels by inducing insulin transcription-related genes, suggesting that glucose metabolism is closely associated with HOTAIR [61]. Li et al. [62] reported upregulation of lncRNA-MIAT in plasma samples of patients with NPDR and patients without diabetic retinopathy, as well as in human adult retinal pigment epithelial cells (ARPE-19) in a high-glucose environment. It was suggested that overexpressed lncRNA-MIAT was associated with diabetic retinopathy and reduced viability of ARPE-19 cells by upregulating TGFβ1. Upregulation of lncRNA KCNQ1OT1 in the serum samples of patients with PDR and NPDR, as well as in human retinal endothelial cells (hRECs), was observed. lncRNA KCNQ1OT1 promoted the expression of EGFR by inhibiting miR-1470. The lncRNA KCNQ1OT1 also plays a role in the proliferation and epithelial-to-mesenchymal transition (EMT) of lens epithelium, thus aiding in the progression of diabetes retinopathy [63].

#### 2.3.3. Downregulated miRNAs in Diabetic Retinopathy

Prado et al. [64] examined differential miR-320a expression in the plasma samples of diabetes patients with and without diabetic retinopathy. Downregulation of miR-320a was observed in patients with diabetic retinopathy. Further, it was reported that miR-320 targeted about 16 genes: *YOD1*, *YWHAE*, *PTEN*, *TSC1*, *CDK6*, *MAPK8IP3*, *IGF2BP3*, *ARPP19* and *FOXM1*, which play roles in the regulation of cellular response to stress, the cell cycle and the negative regulation of the cellular protein metabolic process. *PDX3* (a homolog of *PDX1*) and *GXYLT1* have been implicated in regulating pancreatic development [65,66,67,68]. Li et al. [56] determined the expression of miRNAs from the peripheral serum of 21 T2D patients and reported that miR-485-5p, miR-4448, miR-9-5p and miR-338-3p were suppressed in patients with diabetic retinopathy. It was suggested that these miRNAs can be useful in early diabetic retinopathy risk prediction. Molecular pathway studies and analysis revealed that the aforementioned miRNAs target four genes, viz., *SIRT1*, *FOXO1*, *FOXO3* and *NFKB1*. Target gene prediction revealed that SIRT1 (an important regulator of the revascularization of ischemic tissues) is the potential target of miR-338 and miR-9-5p, whereas *FOXO1* and *FOXO3* (regulating vascular morphogenesis) were targets for miR-485-5p, miR-338-3p and miR-9-5p. It has been reported [69] that miR-423 is negatively associated with the progression of diabetic retinopathy since it is highly downregulated in the serum samples of patients with proliferative diabetic retinopathy.

#### 2.3.4. Downregulated lncRNAs in Diabetic Retinopathy

Downregulation of lncRNA AK077216 was reported in a study [70] conducted using the plasma samples of 60 diabetic retinopathy patients. Downregulated lncRNA AK077216 was observed only in diabetic retinopathy patients as compared to diabetic patients with no complications and controls. miR-383 was negatively associated with lncRNA AK077216 in diabetic retinopathy patients. Subsequently, ARPE-19 cells were used to analyze the expression of lncRNA AK077216 under high-glucose conditions. There was no significant effect of high glucose on lncRNA AK077216 in ARPE-19 cells; however, lncRNA AK077216 was reported to modulate ARPE-19 cell apoptosis by targeting miR-383.

### 2.4. Dysregulated ncRNAs in Diabetic Nephropathy

#### 2.4.1. Upregulated miRNAs in Diabetic Nephropathy

Wu et al. [71] reported significant upregulation of miR-27a-3p in human HK-2 cells treated with high glucose. Further, it was observed that miR-27-3p inhibits the expression of prohibitin and transmembrane BAX inhibitor motif containing 6 (TMBIM6), which play a role in the progression of diabetic nephropathy. Thus, the upregulation of miR-27a-3p is positively associated with diabetic nephropathy via miR-27a-3p-prohibitin/TMBIM6 signaling axis.

#### 2.4.2. Upregulated lncRNAs in Diabetic Nephropathy

LncRNA NR_038323 was overexpressed in high-glucose-induced HK-2 cells, which alleviated HG-induced renal fibrosis by targeting miR-324-3p [72]. It was suggested that miR-324-3p can mediate the high-glucose-induced upregulation of fibronectin, collagen I and collagen IV by targeting dual-specificity protein phosphatase-1 (DUSP1). Thus, lncRNA NR_038323 attenuated renal fibrosis by modulating the levels of fibronectin, collagen I and collagen IV via the miR-324-3p/DUSP1 axis. The study further verified these molecular changes in the human kidney samples of patients with diabetic nephropathy.

#### 2.4.3. Downregulated miRNAs in Diabetic Nephropathy

In a study [73] including 31 diabetic patients (18 with no diabetic complications and 13 with diabetic nephropathy), miR-31 was observed to be significantly downregulated in patients with diabetic nephropathy as compared to patients with no diabetic complications. The presence of diabetic nephropathy involved increased leukocyte adhesion and rolling flux and a lower rolling velocity. Leukocyte rolling velocity was positively associated with miR-31 levels, whereas leukocyte adhesion, as well as TNFα, ICAM-1 and IL-6 levels, were negatively associated with miR-31.

#### 2.4.4. Downregulated lncRNAs in Diabetic Nephropathy

Yang et al. [74] reported the downregulation of long intergenic noncoding RNA for kinase activation (LINK-A) from the renal biopsies of patients with diabetic nephropathy. lncRNA LINK-A was found to be correlated with hypoxia-inducible factor 1α (HIF1α). The activation of HIF1α has been demonstrated to improve diabetic nephropathy [75]. Thus, HIF1α signaling activated by lncRNA LINK-A may improve the treatment of diabetic nephropathy.

### 2.5. Dysregulated ncRNAs in Diabetic Neuropathy

#### 2.5.1. Upregulated miRNAs in Diabetic Neuropathy

Li et al. [76] investigated the dysregulation of miR-199a-3p and reported an overexpression of miR-199a-3p in the serum samples of patients with diabetic neuropathy. It was further noted that upregulated miR-199a-3p caused the inhibition of serine protease inhibitor (SerpinE2). miR-199a-3p induces diabetes neuropathy by promoting coagulation by targeting SerpinE2, which is known to prevent cartilage catabolism.

#### 2.5.2. Upregulated lncRNAs in Diabetic Neuropathy

LncRNA MALAT1 was significantly upregulated in the peripheral-blood mononuclear cells of patients with diabetic neuropathy [77]. Further, a significant positive association between MALAT1 and C-X-C motif chemokine receptor 4 (CXCR4) was observed. It was earlier reported that CXCR4 chemokine receptor signaling mediates pain in diabetic neuropathy [78]. Therefore, the study suggests a possible involvement of the MALAT1/CXCR4 axis in the pathogenesis of diabetic neuropathy.

#### 2.5.3. Downregulated miRNAs in Diabetic Neuropathy

MiR-146a was reported [79] to be downregulated in the PBMCs of T2D patients with diabetic neuropathy. A negative correlation was observed between miR-146a and TNF-α and IL-6 levels. The downregulation of miR-146a was associated with diabetic neuropathy via activation of the NF-κB gene and an increase in the expression of TNF-α and IL-6.

### 2.6. Dysregulated ncRNAs in Gestational Diabetes

#### 2.6.1. Upregulated miRNAs in Gestational Diabetes

Stirm et al. [80] studied the microRNA expression profiles of maternal and fetal WBCs from 30 women with gestational diabetes. It was observed that miR-340 was upregulated and PAIP1, a miR-340 target gene, was downregulated in women with gestational diabetes at the protein and mRNA levels. These results provide evidence for the miRNA-dependent programming of maternal WBCs in gestational diabetes.

#### 2.6.2. Upregulated lncRNAs in Gestational Diabetes

Zhang et al. [81] studied the expression level of lncRNA MALAT1 in placental tissues from 78 gestational diabetes patients and 30 normal pregnant women. It was reported that lncRNA MALAT1 was upregulated in gestational diabetes patients. Further, they investigated the role of lncRNA in gestational diabetes using the placental trophoblastic-derived cell line HTR8, which was cultured in a high-glucose environment. It was observed that lncRNA MALAT1 downregulation in HTR8 cells in high-glucose conditions inhibited the cell proliferation, migration and invasion potency of HTR8. It was also reported that the downregulation of lncRNA MALAT1 led to the suppression of inflammatory factors and led to inhibition of the TGF/NF-κB pathway, suggesting that lncRNA MALAT1 affects the biological behavior of HTR8 through regulation of the TGF/NF-κB pathway.

#### 2.6.3. Downregulated miRNAs in Gestational Diabetes

Sun et al. [82] studied the expression of miR-29b in placental tissues from 204 gestational diabetes patients and reported that miR-29b was downregulated in these patients. It was also observed that the upregulation of miR-29b led to inhibition of the cell proliferation, viability, migration and invasion potency of trophoblastt cells. It was also reported that miR-29b overexpression led to the downregulation of HIF3A, which is the functional target of miR-29b. MiR-29b suppressed the expression of HIF3A by binding to its 3′ UTR region. Thus, miR-29b downregulation may be related to gestational diabetes development by increasing the expression of HIF3A.

#### 2.6.4. Downregulated lncRNAs in Gestational Diabetes

A microarray analysis [83] of lncRNA expression in exosomes found in the umbilical cord blood of 23 patients with gestational diabetes was conducted wherein several lncRNAs, including ENST00000596839.1, lnc-EIF4ENIF1-1:1, lnc-TBC1D30-4:1 and lnc-ZNF800-1:1, were downregulated. miR-362-5p was reported to be a target of lnc-ZNF800-1:1. However, the functional relevance of this regulation is yet to be elucidated.

### 2.7. Network Architecture of Dysregulated ncRNAs in Diabetes

**Figure 1:**
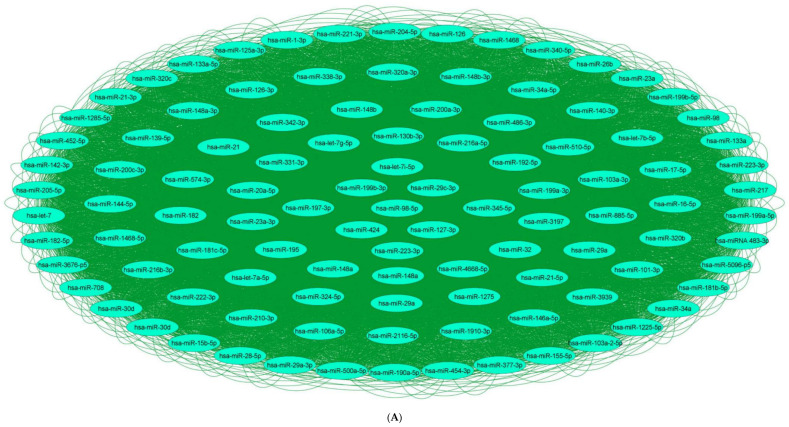

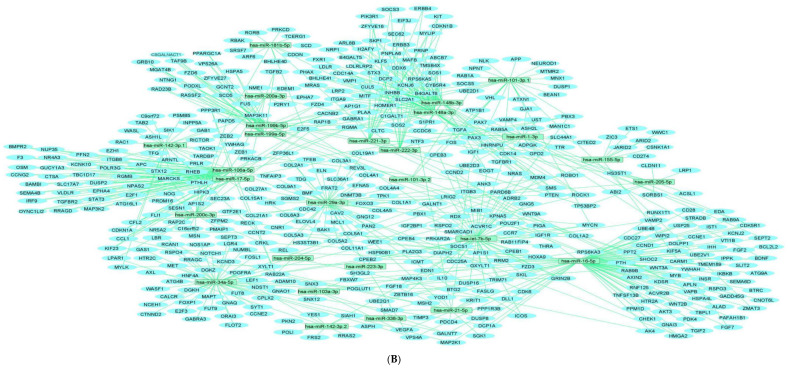
**Upregulated human microRNA networks in diabetes:** (**A**) Human microRNA–microRNA network: Human miRNA–miRNA interaction network with 106 nodes and 11,130 edges. The networks were constructed using Cytoscape 3.9.1 [84]. (**B**) Human microRNA–gene target network: Architecture of networks of upregulated miRNAs implicated in diabetes in humans showing interaction network of functionally enriched miRNAs with their targets, with 484 nodes and 691 edges. The networks were constructed using Cytoscape 3.9.1 [84]. Using Mienturnet [85], the miRNA–target interactions were identified by TargetScan [86], and the functionally enriched miRNAs were generated using the KEGG database [87].

**Figure 2:**
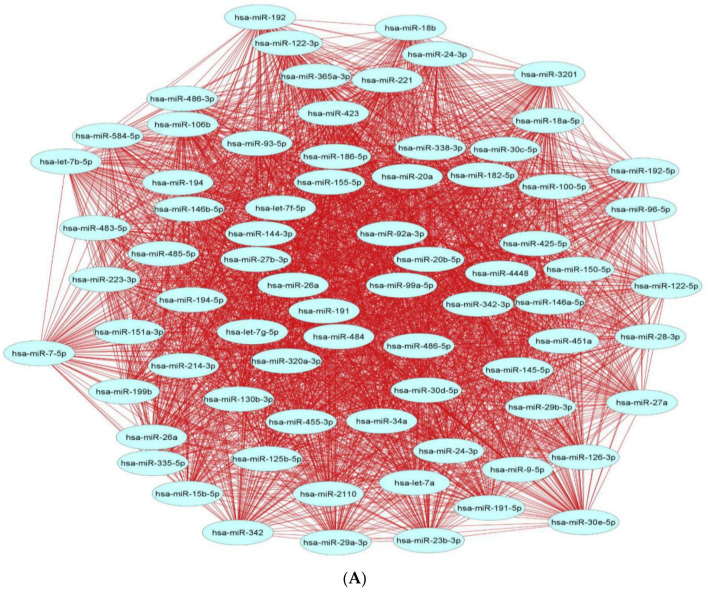

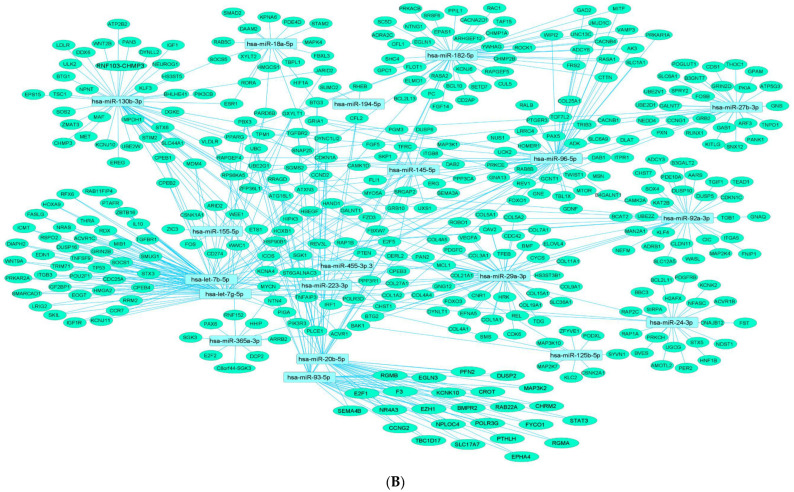
**Downregulated human microRNA networks in diabetes:** (**A**) Human microRNA–microRNA network: Human miRNA–miRNA interaction network with 74 nodes and 2701 edges. The networks were constructed using Cytoscape 3.9.1 [84]. (**B**) Human microRNA–gene target network: Architecture of networks of downregulated miRNAs implicated in diabetes in humans showing interaction network of functionally enriched miRNAs with their targets, with 417 nodes and 583 edges. The networks were constructed using Cytoscape 3.9.1 [84]. Using Mienturnet [85], the miRNA–target interactions were identified by TargetScan [86] and the functionally enriched miRNAs were generated using the KEGG database [87].

**Figure 3:**
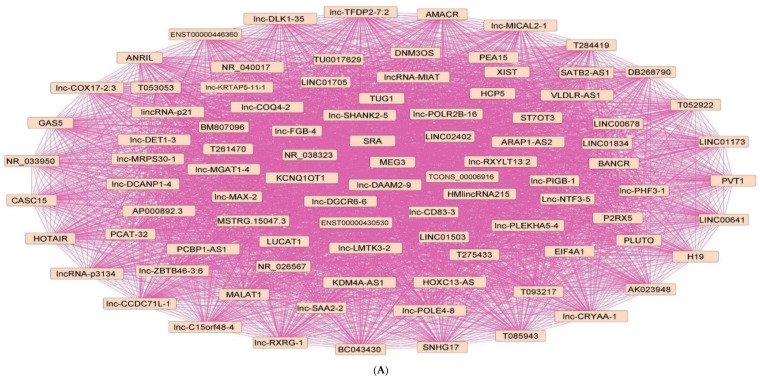

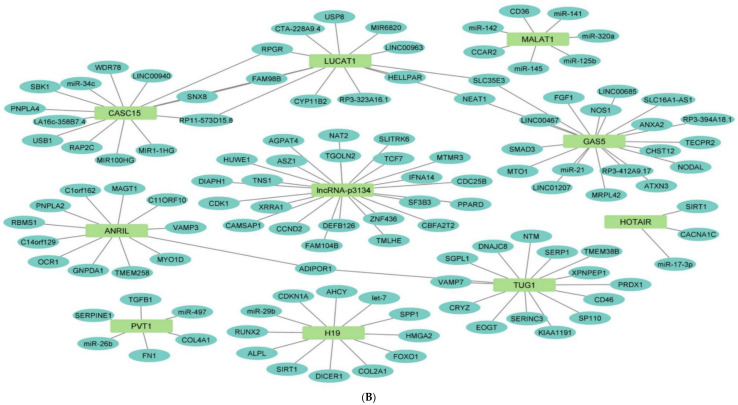
**Upregulated human lncRNA networks in diabetes:** (**A**) Human lncRNA–lncRNA network: Human lncRNA–lncRNA interaction network with 92 nodes and 4186 edges. The networks were constructed using Cytoscape 3.9.1 [84]. (**B**) Human lncRNA–gene target network: Architecture of networks of upregulated lncRNAs implicated in diabetes in humans showing interaction network of lncRNAs with their targets, with 127 nodes and 124 edges. The networks were constructed using Cytoscape 3.9.1 [84]. The lncRNA–target interactions were identified by lncRNA2target v3.0 [93] and LncRRIsearch [94].

**Figure 4:**
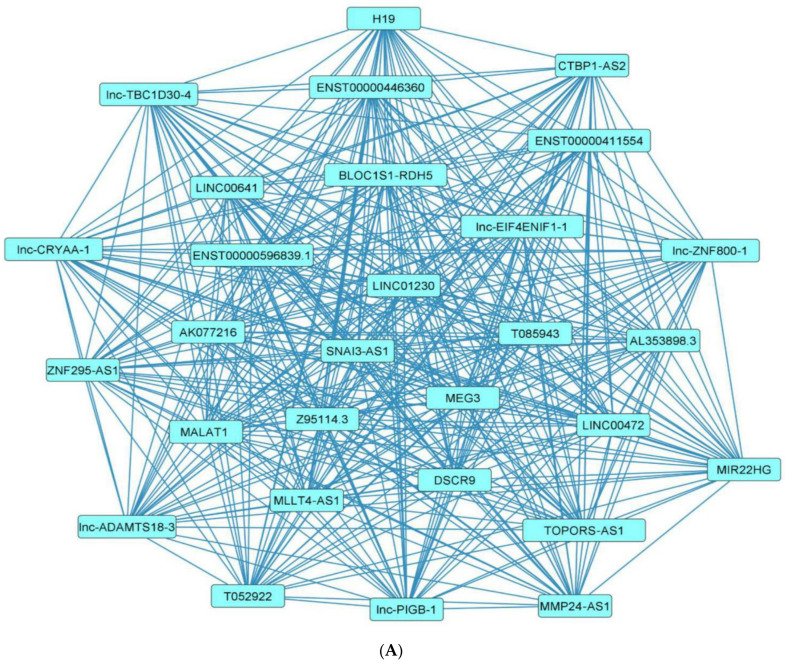

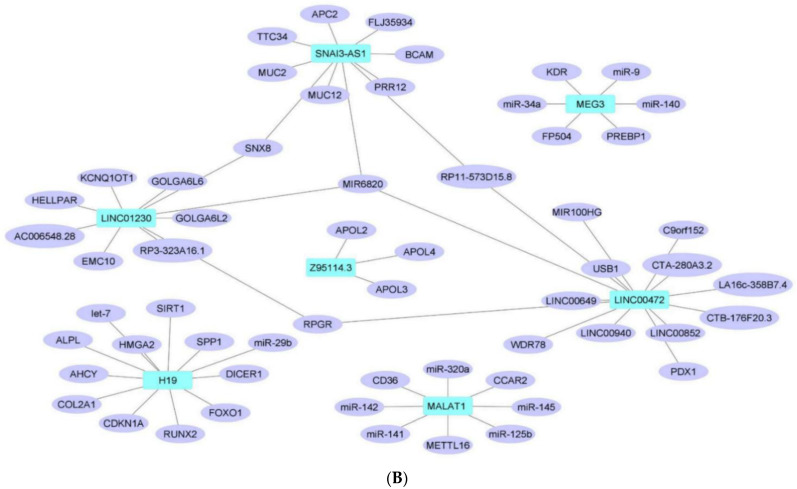
**Downregulated human lncRNA networks in diabetes:** (**A**) Human lncRNA–lncRNA network: Human lncRNA–lncRNA interaction network with 29 nodes and 406 edges. The networks were constructed using Cytoscape 3.9.1 [84]. (**B**) Human lncRNA–gene target network: Architecture of networks of downregulated lncRNAs implicated in diabetes in humans showing interaction network of lncRNAs with their targets with 65 nodes and 63 edges. The networks were constructed using Cytoscape 3.9.1 [84]. The lncRNA–target interactions were identified by lncRNA2target v3.0 [93] and LncRRIsearch [94].

Investigation of the network topology of dysregulated ncRNAs in diabetes is imperative for biomarker discovery. In particular, the delineation of major target hubs based on the degree of the nodes, the directionality of the network (if known) and the presence of network motifs, including feed-forward loops, bifan motifs, etc., can shed tremendous light on novel biomarkers that can be exploited for theranostic and/or preventative intervention in diabetes. We classify dysregulated ncRNA biological networks in diabetes as follows:

(a) *Upregulated human microRNAs:* Upregulated human microRNAs implicated in diabetes are summarized in Table 1. miRNA–miRNA and miRNA–target networks of upregulated human miRNAs in diabetes were constructed using an in silico method, as shown in Figure 1A and 1B, respectively.
(b) *Downregulated human microRNAs:* Downregulated human microRNAs implicated in diabetes are summarized in Table 2. miRNA–miRNA and miRNA–target networks of downregulated human miRNAs in diabetes were constructed using an in silico method, as shown in Figure 2A and 2B, respectively.
(c) *Upregulated human lncRNAs:* Upregulated human lncRNAs implicated in diabetes are summarized in Table 3. lncRNA–lncRNA and lncRNA–target networks of upregulated human lncRNAs in diabetes were constructed using an in silico method, as shown in Figure 3A and 3B, respectively.
(d) *Downregulated human lncRNAs*: Downregulated human lncRNAs implicated in diabetes are summarized in Table 4. lncRNA–lncRNA and lncRNA–target networks of downregulated human lncRNAs in diabetes were constructed using an in silico method, as shown in Figure 4A and 4B, respectively.

**Table 1:** Upregulated human microRNAs in diabetes.

Sr. No.	miRNA	Biological Matrix (Cell Line/Patient)	Targets	References
1	hsa-miR-29a	Serum samples of 18 subjects with newly diagnosed with T2D.	-	[45]
2	hsa-miR-34a	Plasma samples of prediabetic subjects (*n* = 12), and T2D (*n* = 31) and T1D patients (*n* = 16).	-	[46]
3	hsa-miR-1	Serum samples of 78 males with uncomplicated T2D	-	[48]
4	hsa-miR-148b-3p	Plasma samples of 33 T1D mellitus patients	TP53, PTPN1, FADD, IRS2, CXCL8, IL8, PIK3R1, MAPK8/JNK, AKT3, IKBKB/IKKB	[33]
5	hsa-miR-1275			
6	hsa-miR-103a-3p			
7	hsa-miR-200a-3p			
8	hsa-miR-342			
9	hsa-miR-148a-3p			
10	hsa-miR-21-5p			
11	hsa-miR-210-3p			
12	hsa-miR-338-3p			
13	hsa-miR-200c-3p			
14	hsa-miR-155-5p			
15	hsa-miR-320			
16	hsa-miR-21-3p			
17	hsa-miR-340-5p			
18	hsa-miR-133a-5p			
19	hsa-miR-2116-5p	Serum samples of 45 patients with diabetic retinopathy	NOTCH2	[57]
20	hsa-miR-142-3p	Isolated human CD4+ T cells from serum samples of children with and without islet autoimmunity	TET2	[40]
21	hsa-miR-23a	Serum samples of 22 children at onset of T1D	-	[38]
22	hsa-miR-98			
23	hsa-miR-197-3p	Plasma samples of 40 children diagnosed with new-onset T1D	TUSC2, NSUN5, CD82, BMF, PMAIP1, MTHFD1	[39]
24	hsa-miR-199a-3p	Plasma samples of 60 patients with T2D; lower limb skin samples from 30 patients with diabetic neuropathy	SERPINE2	[76]
25	hsa-miR-20a-5p	Serum samples of T2D patients with diabetic retinopathy	-	[58]
26	hsa-miR-223-3p			
27	hsa-let-7a-5p			
28	hsa-miR-885-5p	Serum samples of 455 type 1 diabetic patients	-	[35]
29	hsa-miR-486-3p			
30	hsa-miR-574-3p			
31	hsa-miR-140-3p			
32	hsa-miR-17			
33	hsa-miR-222			
34	hsa-miR-16			
35	hsa-miR-106a			
36	hsa-miR-139-5p			
37	hsa-miR-210	Plasma samples of 54 type 2 diabetic patients without coronary artery disease and 46 type 2 diabetic patients with coronary artery disease	-	[49]
38	hsa-miR-190a-5p	Serum samples of 21 T2D patients	-	[56]
39	miR-27a-3p	Human HK-2 cells	Prohibitin, TMBIM6	[71]
40	miR-340	Maternal and fetal WBCs from 30 women with gestational diabetes	PAIP1	[80]

**Table 2:** Downregulated human microRNAs in diabetes.

Sr. No.	miRNA	Biological Matrix (Cell Line/Patient)	Targets	References
1	hsa-miR-130b	Serum samples obtained from 327 T2D patients	-	[52]
2	let-7b	Whole peripheral blood from 40 T2D patients	IGF2BP2	[53]
3	hsa-miR-320a	Plasma samples of 48 diabetes patients without diabetic retinopathy and 62 diabetes patients with diabetic retinopathy	FOXM1, YWHAZ, GTPBP2, YOD1, YWHAE, TSC1, CPD, PBX3, MAPK8IP3, GXYLT1, ARPP19, SYNGR2, CDK6, IGF2BP3, PTRN, KITLG	[64]
4	hsa-miR-214-3p	Whole-blood samples from 40 T2D patients	MAP2K5, CDK6, RASA1, MAPK14, FLOT1, MPL, IL13, ACVR1B, TGFB1, NOS3, PTPRF, ATF4, TRIB3, FGFR3	[54]
5	hsa-miR-27b-3p			
6	miR-23b-3p			
7	miR-24-3p			
8	miR-29b-3p			
9	miR-451a			
10	let-7f-5p			
11	miR-34a	Serum samples of 18 subjects newly diagnosed with T2D and 19 pre-diabetic individuals	-	[45]
12	miR-4448	Serum samples of 21 T2D patients	-	[56]
13	miR-9-5p			
14	miR-338-3p			
15	hsa-miR-485-5p			
16	miR-423	Serum samples of 10 T2D patients without retinopathy, 22 with non-proliferative diabetic retinopathy and 15 with proliferative diabetic retinopathy	-	[69]
17	hsa-miR-155	Serum samples of 455 type 1 diabetic patients	-	[35]
18	hsa-miR-92a			
19	hsa-miR-483-5p			
20	hsa-miR-29a			
21	hsa-miR-320			
22	hsa-miR-145			
23	hsa-miR-146a			
24	hsa-miR-191			
25	hsa-miR-342			
26	hsa-miR-223			
27	hsa-miR-24			
28	hsa-miR-150			
29	miR-484			
30	miR-486-5p			
31	hsa-miR-126	Plasma samples of 54 T2D patients without coronary artery disease and 46 type 2 diabetic patients with coronary artery disease	-	[49]
32	miR-31	Serum samples of 31 diabetic patients (18 with no diabetic complications and 13 with diabetic nephropathy)	TNFα, ICAM-1, IL-6	[73]
33	miR-146	PBMCs of T2D patients with diabetic neuropathy	TNF-α, IL-6, NF-κB	[79]
34	miR-29b	Placental tissues from 204 gestational diabetes patients	HIF3A	[82]

**Table 3:** Upregulated human lncRNAs in diabetes.

Sr. No.	lncRNA	Biological Matrix (Cell Line/Patient)	Targets	References
1	lncRNA-p3134	Whole-blood samples of 30 T2D patients	TNS1, ASZ1, DIAPH1, IFNA14, ZNF436, MTMR3, CDK1, PPARD, TCF7, HUWE1, CCND2	[50]
2	ANRIL	Peripheral-blood mononuclear cells (PBMCs) from 32 patients with T2D	-	[51]
3	ENST00000550337			
4	GAS5			
5	HOTAIR			
6	lincRNA-p21			
7	PLUTO			
8	HOTAIR	Serum samples of 30 diabetic patients, 30 with non-proliferative diabetic retinopathy (NPDR) and 20 with retinopathy (PDR)	-	[59]
9	MALAT1			
10	lncRNA-MIAT	Plasma samples of 52 patients with NPDR and ARPE-19 cells	TGFβ1	[62]
11	KCNQ1OT1	Serum samples of 20 patients with proliferative diabetic retinopathy and 20 with non-proliferative diabetic retinopathy, human retinal endothelial cells (hRECs)	miR-1470, EGFR	[63]
12	BANCR	Plasma samples of 64 patients with diabetic retinopathy, human retinal pigment epithelial cell line ARPE-19	-	[88]
13	HEIH	Serum samples of 36 patients with diabetic retinopathy, human adult retinal pigment epithelial cells (ARPE-19)	miR-939	[89]
14	CASC15	Plasma samples of 50 diabetic patients with chronic renal failure	miR-34c	[90]
15	lnc-COX17-2:3	Differentially expressed lncRNAs in umbilical cord-blood exosomes of 23 gestational diabetes patients	-	[83]
16	lnc-ZBTB46-3:6			
17	lnc-RXYLT13:2			
18	lnc-TFDP2-7:2			
19	AK023948	Plasma samples from 25 patients with T1D	-	[41]
20	BC043430			
21	DGCR5			
22	MEG3			
23	PCAT-32			
24	SRA			
25	ST7OT3			
26	TU0017629			
27	LincRNA-VLDLR			
28	H19	Plasma samples obtained from 30 patients with diabetes mellitus, human umbilical vein endothelial cells (HUVECs)	miR-29b	[91]
29	DNM3OS	CD14+ monocytes obtained from T2D patients	ILF-2	[92]
30	SRA	Plasma samples of 25 patients with T1D	miR-146b	[41]
31	NR_038323	Human HK-2 cells	miR-324-3p	[72]
32	MALAT1	PBMCs of patients with diabetic neuropathy	CXCR4	[77]
33	MALAT1	Placental tissues from 78 gestational diabetes patients and 30 normal pregnant women	TGF, NF-κB	[81]

**Table 4:** Downregulated human lncRNAs in diabetes.

Sr. No.	lncRNA	Biological Matrix (Cell Line/Patient)	Targets	References
1	CTBP1-AS2	Peripheral-blood mononuclear cell (PBMC) of 100 type 2 diabetic patients	TGF-β	[55]
2	AK077216	Plasma samples from 60 diabetic retinopathy patients; human adult retinal pigment epithelial cells (ARPE-19)	miR-383	[70]
3	ENST00000596839.1	Differentially expressed lncRNAs in umbilical cord-blood exosomes of 23 gestational diabetes patients	miR-362-5p	[83]
4	lnc-EIF4ENIF1-1:1			
5	lnc-TBC1D30-4:1			
6	lnc-ZNF800-1:1			
7	LINK-A	Renal biopsies of patients with diabetic nephropathy	HIF1α	[74]

## 3. Spatial Dysregulation of ncRNAs in Diabetes

Some ncRNAs in diabetes are spatially dysregulated, i.e., in tissues, mesenchymal stem cells, pancreatic beta cells, etc. These ncRNAs cannot directly serve as biomarkers for diabetes as they cannot be detected in plasma samples. However, spatially dysregulated ncRNAs in diabetes may help in providing better insights into novel therapeutic targets and prognostic markers for management of the disorder. In this section we discuss a few examples of ncRNAs implicated in diabetes that are spatially dysregulated.

The spatially dysregulated ncRNAs implicated in diabetes are summarized in Table 5.

### 3.1. Pancreatic Beta Cells

Santos et al. [95] investigated the serum miR-101-3p expression of recent-onset patients with T1D and observed that it caused inhibition of insulin regulation and cell survival while being highly upregulated in pancreatic β cells. It was also inferred that ephrin receptor, mesenchymal–epithelial transition factor-hepatocyte growth factor (c-Met-HGF), and signal transducer and activator of transcription 3 (STAT3)—associated with insulin production and survival, as well as the death of pancreatic β cells—are the targets of miR-101-3p. Further, the promotion of pancreatic differentiation in human induced pluripotent stem cells (hiPSCs) from human foreskin fibroblasts by miR-375 was demonstrated [96]. Islet-like cells were obtained by overexpressing miR-375 in hiPSCs, which secreted insulin in a glucose-dependent manner.

### 3.2. Mesenchymal Stem Cells

Using human mesenchymal stem cells (MSCs), Zhu et al. [97] studied the dysregulation of miR-205-5p, which was highly overexpressed in MSCs, improving the therapeutic application of MSCs in diabetic foot. In addition, lncRNA MALAT1 was reported [98] to be downregulated in human bone marrow-derived MSCs. MALAT1 overexpression resulted in the suppression of miR-205-5p, which led to a significant increase in VEGF cellular protein, promoting a VEGF-mediated therapeutic effect on diabetic foot (DF). There was no significant effect on VEGF mRNA by the upregulation of MALAT1 or suppression of miR-205-3p; thus, MALAT1 appeared to act as a VEGF post-transcriptional activator by inhibiting miR-205-3p. Further, upregulated MALAT1 also improved endothelial cell tube formation in vitro. Interestingly, Wei et al. [99] studied the effects of extracellular vesicles (EVs) secreted by human umbilical cord mesenchymal stem cells (hucMSCs) on angiogenesis and wound healing under hyperglycaemic conditions in vivo and in vitro. Local application of hucMSC-EVs in vivo promoted angiogenesis and diabetic wound healing. HucMSC-EVs promoted migration, proliferation, and tube formation by inhibiting phosphatase and tensin homolog (PTEN) expression and activating the AKT/HIF-1α/VEGF pathways in vitro. HucMSC-EVs were reported to be highly enriched with miR-17-5p. MiR-17-5p agomirs suppressed PTEN expression, resulting in AKT/HIF-1α/VEGF pathway activation to promote migration, proliferation, and tube formation in HG-treated human umbilical vein endothelial cells (HUVECs) in vitro. miR-17-5p agomirs mimicked the effects of hucMSC-EVs on wound healing and angiogenesis in vivo, whereas their effects were reversed by miR-17-5p inhibitors. The study further suggested that hucMSC-EVs have protective and regenerative effects on HG-induced endothelial cells via the transfer of the miR-17-5p targeting PTEN/AKT/HIF-1α/VEGF pathways, leading to enhanced diabetic wound healing. miR-375 was transfected in adipose-derived stem cells (ADSCs) isolated from the adipose tissue of diabetic patients [100]. It was observed that after the treatment of ADSCs with lentiviruses containing miR-375, islet-like cluster (ILC)-specific genes such as pancreatic and duodenal homeobox 1 (PDX1) and insulin were highly upregulated. The study concluded that ILCs can be achieved by overexpressing miR-375 in ADSCs. Finally, Li et al. [101] studied the role of miR-181c-5p in promoting insulin-producing cell (IPC) synthesis from hiPSCs by targeting Smad7 and TGIF2.

### 3.3. Human Retinal Microvascular Endothelial Cells (hRMECs)

Upregulation of MALAT1 was reported [102] in hRMECs treated with high glucose. MALAT1 competitively bound to miR-125b, which promoted the expression of VE-cadherin. Inhibiting MALAT1 resulted in suppression of the VE-cadherin/β-catenin complex by targeting miR-125b. This suggests that MALAT1 may serve as a promising target in anti-angiogenic therapy for DR.

### 3.4. Human Lens Epithelial Cells

LncRNA KCNQ1OT1 was upregulated and miR-26a-5p was downregulated in diabetic cataract posterior lens-capsule tissues and high-glucose (HG)-treated lens epithelial cells SRA01/04 [103]. The inhibition of lncRNA KCNQ1OT1 or upregulation of miR-26a-5p suppressed cell viability as well as migration and epithelial–mesenchymal transition (EMT) in HG-treated SRA01/04 cells. lncRNA KCNQ1OT1 modulated migration, cell viability and EMT by targeting miR-26a-5p. Integrin alpha-V (ITGAV) was targeted by miR-26a-5p, while lncRNA KCNQ1OT1 promoted the expression of ITGAV. The overexpression of ITGAV promoted cell viability, migration and EMT in HG-treated SRA01/04 cells, whereas lncRNA KCNQ1OT1 silencing mitigated these effects. Further, lncRNA KCNQ1OT1 knockdown suppressed the high-glucose-induced promotion of TGF-β/Smad3 signaling by modulating miR-26a-5p [103].

### 3.5. Human Adult Retinal Pigment Epithelial (ARPE-19) Cells

In a study by Tong et al. [104] using ARPE-19, high glucose levels promoted the expression of miR-34a five-fold with the downregulation of MEG3 and SIRT1. The study showed that upregulation of MEG3 caused the inhibition of miR-34a and the upregulation of SIRT1. MEG3 could alleviate diabetic retinopathy by forming a competing endogenous RNA (ceRNA) network with miR-34a. In the same study, differentially expressed lncRNA MEG3 was investigated in ARPE-19 cells. It was observed that high glucose significantly inhibited MEG3 as well as SIRT1 expression, while miR-34a was highly overexpressed. MEG3 was reported to promote the expression of SIRT1 by sponging miR-34a. Overexpressed MEG3 and miR-34a inhibition alleviated the effect of high-glucose-induced apoptosis in ARPE-19 cells. MEG3 upregulation inhibited the expression of inflammatory cytokines such as TNF-α, IL-1β and IL-6, alleviating high-glucose (HG)-induced inflammation. IκB, which is a key regulator of NF-κB, was decreased along with SIRT1 in high-glucose conditions, suggesting that the NF-κB signaling pathway could be activated in high-glucose conditions. However, MEG3 overexpression impeded NF-κB signaling by reversing the expression of IκB and SIRT1; thus, MEG3 could suppress NF-κB signaling via the miR-34a/SIRT1 axis. The expression of the apoptosis-related protein Bax was upregulated, while expression of the Bcl-2 protein was suppressed in ARPE-19 cells under high-glucose conditions. High-glucose conditions caused a decrease in the Bcl-2/Bax ratio, leading to apoptosis. MEG3 could alleviate these effects by regulating the Bcl-2/Bax ratio via the miR-34a/SIRT1 axis.

### 3.6. Liver Cells and Diabetic Patient Livers

miR-223 regulated insulin resistance by targeting FOXO1 in a study using HepG2 cells [105]. Upregulation of miR-223 caused suppression of the FOXO1 protein, leading to insulin resistance. In another study, it was reported [106] that downregulation of miR-26a in the liver samples of patients with T2D caused impaired insulin sensitivity along with hyperglycemia. Further, it was inferred that miR-26a plays a critical role in regulating hepatic metabolism.

### 3.7. Human Umbilical Vein Endothelial Cells (HUVEC)

Silambarasan et al. [107] investigated the dysregulation of miRNAs in HUVECs predisposed to various levels of glucose concentrations. It was reported that miR-192-5p, miR-221-3p and miR-29c-3p were upregulated under hyperglycemic conditions. Glucose-induced endothelial dysfunction was positively associated with the upregulation of these miRNAs.

### 3.8. Human Kidney-2 (HK-2) Cells

Meng et al. [108] used high-glucose-treated HK-2 cells to examine the dysregulation of miR-216a-5p. High-glucose upregulated miR-216a-5p, restoring the suppressive effect of lncRNA ZEB1-AS1 upregulation on the epithelial-to-mesenchymal transition and fibrogenesis.

### 3.9. Preretinal Fibrovascular Membranes

A study [109] including 20 Han Chinese diabetic patients with PDR who underwent pars plana vitrectomy was conducted to evaluate the effect of intravitreal conbercept (VEGF inhibitor) injection on the expression of lncRNAs. The fibrovascular membranes of these patients were harvested during surgery to evaluate the expression of lncRNAs. Several lncRNAs, including ENST00000446360, ENST00000569661, NR_038970, T052922, T085943 and TCONS_00029045, were reported to be downregulated (fold change > 2), and the lncRNAs ENST00000446360, ENST00000532530, ENST00000553754, ENST00000569661, NR_026567, NR_038970, T048173, T052922, T053053, T085943, T093217, T103904, T275433, T284419, TCONS_00029045 and TCONS_00020046 were reported to be upregulated. It was suggested that conbercept injection caused a remarkable change in lncRNA expression when compared with the control group.

### 3.10. Human Dermal Microvascular Endothelial Cells (HMEC-1)

Tao et al. [110] reported significant downregulation of lncRNA-H19 in HMEC-1 under hyperglycemic conditions. The inhibition of lncRNA-H19 resulted in impairment of the insulin–phosphatidylinositol 3-kinase (PI3K)–Akt pathway, resulting in impaired angiogenesis and wound healing in diabetes.

**Table 5:** Spatial dysregulation of ncRNAs in diabetes.

Sr. No.	ncRNA	Biological Matrix (Cell Line/Patient)	Targets	Upregulated/ Downregulated	References
1	hsa-miR-452-5p	Human umbilical vein endothelial cell (HUVEC) samples isolated from pregnant women with gestational diabetes mellitus and normal glucose tolerance; placenta samples from women with gestational diabetes mellitus; BeWo cells initiated from malignant gestational choriocarcinoma of the fetal placentae	-	Upregulated	[111]
2	hsa-miR-34a	Human adult retinal pigment epithelial cells (ARPE-19)	SIRT1	Upregulated	[104]
3	microRNA-205-5p	Human mesenchymal stem cells (MSCs)	VEGF	Upregulated	[97]
4	hsa-miR-223	HepG2 human hepatic carcinoma cells	FOXO1	Upregulated	[105]
5	hsa-miR-192-5p	Human umbilical vein endothelial cells (HUVECs)	BCL2, MCL1	Upregulated	[107]
6	hsa-miR-221-3p				
7	hsa-miR-29c-3p				
8	hsa-miR-26b	Articular cartilage obtained from primary osteoarthritis patients, including 20 patients with diabetes	CTGF	Upregulated	[112]
9	hsa-miR-216a-5p	Kidney tissues from kidney biopsies of 20 patients with diabetic nephropathy; human kidney 2 (HK-2) cells	BMP7	Upregulated	[108]
10	hsa-miR-181c-5p	Human induced pluripotent stem cells (hiPSCs)	TGIF2, Smad7	Upregulated	[101]
11	miR-26a	Human hepatoma-derived HuH-7 cells, human liver samples from patients with T2D	-	Downregulated	[106]
12	ENST00000446360	Preretinal fibrovascular membranes harvested from 20 patients diagnosed with proliferative diabetic retinopathy who underwent pars plana vitrectomy	-	Upregulated	[109]
13	ENST00000532530				
14	ENST00000553754				
15	ENST00000569661				
16	NR_026567				
17	NR_038970				
18	T048173				
19	T052922				
20	T053053				
21	T085943				
22	T093217				
23	T103904				
24	T275433				
25	T284419				
26	TCONS_00029045				
27	TCONS_00020046				
29	MALAT1	Human retinal microvascular endothelial cells (hRMECs)	miR-125b, VE-cadherin	Upregulated	[102]
30	MALAT1	HK-2 (human kidney 2) cells	miR-145	Upregulated	[113]
31	NR_047507	Human umbilical vein endothelial cells (HUVECs)	-	Upregulated	[114]
32	NR_125792				
33	T261470				
34	BM807096	HK-2 (human kidney 2) cells	miR-324-3p	Upregulated	[72]
35	DB268790				
36	ENST00000418149				
37	ENST00000429530				
38	ENST00000430530				
39	ENST00000442326				
40	ENST00000452283				
41	ENST00000490856				
42	ENST00000511712				
43	ENST00000511962				
44	ENST00000527083				
45	ENST00000556637				
46	ENST00000567374				
47	HMlincRNA215-				
48	NR_026830				
49	NR_033950				
50	NR_038323				
51	NR_040017				
52	TCONS_00006916				
53	TCONS_00011702				
54	TCONS_00020354				
55	XIST	Renal tissues of 53 patients with diabetic nephropathy, HK-2 (human kidney 2) cells	miR-93-5p	Upregulated	[115]
56	MSTRG.15047.3	Human retinal endothelial cells (hRECs), serum samples and aqueous humor from 30 diabetic retinopathy patients	PBRM1, FGF13	Upregulated	[116]
57	AC008403.3				
58	PVT1	Human chondrocytes isolated from knee cartilage of 20 osteoarthritis patients with and without diabetes	miR-26b	Upregulated	[112]
59	LUCAT1	AC16 cardiomyocyte cells	CYP11B2	Upregulated	[117]
60	ARAP1-AS2	HK-2 (human kidney 2) cells	CDC42	Upregulated	[118]
61	H19	Human dermal microvascular endothelial cells (HMEC-1) and human embryonic kidney cells 293 (HEK293)	-	Downregulated	[110]
62	MEG3	Human adult retinal pigment epithelial cells (ARPE-19)	miR-34a	Downregulated	[104]
63	ENST00000446360	Preretinal fibrovascular membranes harvested from 20 patients diagnosed with proliferative diabetic retinopathy who underwent pars plana vitrectomy	-	Downregulated	[109]
64	ENST00000569661				
65	NR_038970				
66	T052922				
67	T085943				
68	TCONS_00029045				
69	MALAT1	Human umbilical vein endothelial cells (HUVEC); human bone marrow-derived mesenchymal stem cells (MSCs)	miR-205-5p	Downregulated	[98]
70	hsa-miR-101-3p	Serum samples of 50 patients with recent-onset diabetes	HGF, MET proto-oncogene, GAB1, RAC1, COX2, c-FOS	Upregulated	[95]
71	miR-375	Human induced pluripotent stem cells (hiPSCs)	-	Upregulated	[96]
72	miR-17-5p	Human umbilical cord mesenchymal stem cells (hucMSCs)	PTEN	Upregulated	[99]
73	miR-375	Adipose-derived stem cells (ADSCs)	-	Upregulated	[100]
74	KCNQ1OT1	Diabetic cataract posterior lens-capsule tissues and high-glucose (HG)-treated lens epithelial cells SRA01/04	miR-26a-5p	Upregulated	[103]
75	miR-26a-5p	Diabetic cataract posterior lens-capsule tissues and high-glucose (HG)-treated lens epithelial cells SRA01/04	ITGAV	Downregulated	[103]

## 4. ncRNAs as Prognostic Markers in Diabetes

Conserva et al. [119] assayed miRNA regulation in the kidney biopsy samples of patients with diabetic nephropathy (DN) and identified two miRNAs (miR-27b-3p and miR-1228-3p) that were differentially expressed in the biopsies, as well as urine samples, of six diabetic nephropathy patients. Interestingly, both miRNAs were also able to be differentiated among various degrees of renal fibrosis. It was also reported that the urinary expression of miR-27b-3p and miR-1228-3p could be differentiated between diabetic nephropathy patients and glomerulonephritis in diabetic patients. The study concluded that miR-27b-3p and miR-1228-3p can be potential biomarkers for tubular-interstitial fibrosis in patients with DN. Moreover, miR-21 was reported to be a promising biomarker in diabetic cardiomyopathy [120]. A study including 266 subjects with T2D was conducted to assess the diagnostic efficacy of miR-21. It was reported that miR-21, when compared to other diagnostic parameters such as serum hemoglobin A1c (HbA1c), showed higher diagnostic efficiency. In another study including 200 T2D patients, it was observed that miR-29a and miR-29b were highly downregulated (fold changes of 5.62 and 5.58, respectively) [22]. Further, it was reported that the downregulation of these miRNAs might be associated with the severity of T2D in patients.

In a study by Jiménez-Lucena et al. [121] consisting of 462 participants, it was reported that miR-30a-5p and miR-150, as well as miR-375 and miR-15a, were dysregulated in plasma several months before the diagnosis of T2D. The study concluded that these miRNAs could be useful in evaluating the risk of developing diabetes, potentially leading to improved prediction and prevention among people at high risk of T2D. It has been observed [122] that the miRNA miR-4449 is highly upregulated in patients with diabetic nephropathy. The study also reported the involvement of miR-4449 with MAPK signaling, the regulation of AP-1 transcription factor, and integrin function in angiogenesis. Interestingly, it was also proportionately associated with the degree of albuminuria. Thus, miR-4449 may be a putative prognostic and diagnostic marker for diabetic nephropathy. Liu et al. [123] examined the regulation of the miRNAs miR-21, miR-46a, miR-25 and miR-181a in 29 patients with autoimmune diabetes (T1D and latent autoimmune diabetes) compared to 16 type 2 diabetes patients and healthy volunteers. All four miRNAs were significantly suppressed in the serum samples of patients with autoimmune diabetes. Thus, the study concluded that miR-146a and miR-25 may act as putative biomarkers for autoimmune diabetes.

## 5. Signal Transduction with Diabetes-Associated Noncoding RNAs

**Figure 5:**
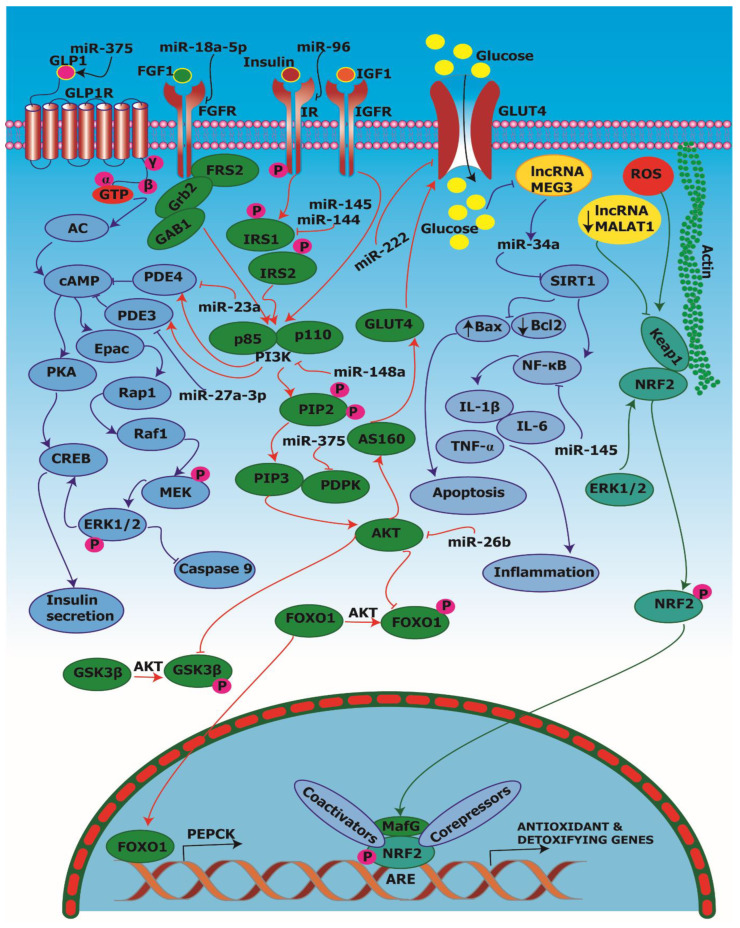
**Signal transduction with diabetes-associated noncoding RNAs:** (i) GLP1 binds to GLP-1 receptor and activates cAMP signaling pathway; miR-375 upregulates GLP 1 ligand. cAMP recruits Epac and PKA to activate CREB, leading to insulin secretion; cAMP is inhibited by PDE3 and PDE4, which leads to reduction in insulin secretion; miR-23a and miR-27a-3p inhibit PDE4 and PDE3, respectively, thus upregulating cAMP. (ii) Insulin binds to the insulin receptor (IR) to activate insulin signaling pathway; insulin receptor is inhibited by miR-96. Upregulation of IR leads to activation of IRS1, PI3K, PIP3/PDPK and AKT, which are, respectively, inhibited by miR-145/miR-144, miR-148a, miR-375 and miR-26b. Activated AKT facilitates translocation of GLUT4 from cytoplasm to cell membrane; miR-222 inhibits GLUT4. AKT mediates inhibitory phosphorylation of GSK3B and FOXO1. FOXO1 translocates to the nucleus and stimulates transcription of PEPCK in the gluconeogenesis pathway. (iii) FGF1 binds to FGF receptor and recruits FRS2, Grb2 and GAB1, which activates the PI3K/AKT pathway, leading to GLUT4 translocation from cytosol to cell membrane. (iv) High glucose concentration inhibits lncRNA MEG3, which, in turn, upregulates miR-34a. Upregulated miR-34a suppresses SIRT1, which leads to decrease in Bcl-2/Bax ratio, leading to apoptosis; suppressed SIRT1 promotes secretion of inflammatory cytokines (IL-6, IL-1β and TNF-α) via NF-κB signaling, leading to diabetic retinopathy. (v) NRF2 is sequestered in the cytoplasm by Keap1 bound to the actin cytoskeleton. Downregulated lncRNA MALAT1 leads to the suppression of inhibitor Keap1 and dissociation of NRF2. ERK1/2 phosphorylates NRF2, and NRF2 translocates to the nucleus where it binds with the antioxidant response element (ARE), stimulating transcription of antioxidant and detoxifying genes.

Herein, we discuss signal transduction pathways involving specific ncRNAs, including miRNAs and lncRNAs, involved in key regulatory roles in the population with diabetes and associated complications. The modulation of signaling pathways by ncRNAs in diabetes is summarized in Figure 5 for the benefit of the reader.

### 5.1. miR-144

Karolina et al. [124] examined the role of miR-144 in insulin signaling in 3T3L1 adipocytes. Circulating miR-144 found in the peripheral blood may function as a biomarker to reflect metabolic disorders. It was reported that elevated levels of miR-144 downregulated mRNA and the protein expression of insulin receptor substrate 1 (IRS1), a key regulator of insulin signaling. In T2D, the level of miR-144 was highly upregulated in pancreas, adipose and liver tissues. IRS1 was inhibited by miR-144, leading to inhibition of the PI3K/AKT pathway.

### 5.2. miR-26b

Xu et al. [125] determined the role of the overexpression, as well as the inhibition, of miR-26b on glucose uptake in the human preadipocytes-visceral (HPA-V) cell line. It was reported that the overexpression of miR-26b led to increased uptake of insulin-stimulated glucose and insulin-stimulated GLUT4 as well as the enhanced serine phosphorylation of AKT. Thus, miR-26b regulated the PI3K/AKT pathway in adipocytes.

### 5.3. miR-96

Yang et al. [126] studied the expression of miR-96 on human hepatocellular carcinoma HepG2 cells. It was reported that the upregulation of miR-96 led to downregulation of the insulin receptor (INSR) as well as the repression of IRS-1 at the post-transcriptional level in hepatocytes. Further, the overexpression of miR-96 led to the alleviation of hepatic insulin signaling and glycogen metabolism due to the inhibition of INSR and IRS-1.

### 5.4. miR-145

Wen et al. [127] studied the expression of miR-145 in hepatocellular carcinoma (HepG2) and human embryonic kidney (HEK293A) cell lines. It was reported that miR-145 expression was upregulated by resistin (adipocyte-derived cytokine) via the p65 pathway. It was also found that miR-145 downregulated IRS-1 at the translational level. In a study [56] on the THP-1 human monocytic leukemia cell line investigating the expression of miR-145 on the stimulation of cytokines, it was reported that the upregulation of miR-145 reduced the expression of the cytokines that included TNF-α, IL-1β and IL-6, as well as reducing the phosphorylation of p65 in the NF-κB pathway in the cell line.

### 5.5. miR-375

Ouaamari et al. [128] studied the expression of miR-375 on the insulinoma-IE (INS-IE) cell line from β-cells. The study showed the interaction between miR-375 and phosphoinositide-dependent kinase-1 (PDPK1). It was reported that miR-375 led to the downregulation of the PDPK1 protein in β-cells, which resulted in a decrease in insulin stimulation. It was also reported that miR-375 led to a decrease in glucose-induced gene expression by affecting the PI3-kinase signaling pathway.

### 5.6. miR-222

In a study conducted by Shi et al. [129] on the expression of miR-222 on omental adipose tissue which was taken from the GDM patients, it was reported that miR-222 was upregulated in omental tissue, which led to the downregulation of estrogen receptor-α (ER-α) and GLUT4 in GDM omental tissue. Thus, it caused saturation of glucose in the blood, resulting in diabetes.

### 5.7. miR-148a

Xu et al. [130] studied the miR-148a expression on the human PTC (papillary thyroid cancer)-derived TPC (thyroid papillary carcinoma) cell line. It was reported that the overexpression of miR-148a led to the suppression of STAT3, PI3K and AKT phosphorylation in PTC. Thus, miR-148a suppressed cell growth PTC through the STAT3 and PI3K/AKT signaling pathways.

### 5.8. miR-18a-5p

The effect of miR-18a-5p on vascular growth factor, such as FGF1 expression, was studied [131] using human retinal microvascular endothelial cells (HRMECs). miR-18a-5p led to suppression of the migration, proliferation and tube formation ability of HRMEC. A reduction in FGF1 during the upregulation of miR-18a-5p at mRNA, as well as at the protein level, was reported. This inhibition of FGF1 led to inhibition of the PI3K/AKT pathway and, thus, GLUT4 translocation was inhibited.

### 5.9. miR-27a-3p

The expression of miR-27a-3p in phosphodiesterase 3 (PDE3) (a cAMP-degrading enzyme) was studied [132] using human cerebral microvascular endothelial cells (hCMECs). It was reported that increased levels of miR-27a-3p led to the suppression of PDE3, which, in turn, enhanced cAMP signaling. Further, the cAMP pathway led to insulin secretion.

### 5.10. miR-23a

Phosphodiesterase 4 (PDE4) can activate and cause inflammation in neuroglia, leading to painful diabetic neuropathy. The activity of miR-23a in the PDE4 enzyme was studied [133] using bupivacaine on C57BL/6J mice. It was reported that bupivacaine upregulated miR-23a expression, which led to the downregulation of PDE4B at the mRNA and protein levels by binding to its 3′-UTR.

### 5.11. lncRNA MALAT1

Highly conserved lncRNA MALAT1 plays a key role in diabetes-associated complications, including diabetic retinopathy (DR). Using human endothelial cells of the retina (HRECs), the role of MALAT1 and its interactions with the kelch-like ECH-associated protein 1/nuclear factor E2-related factor 2 (Keap1/NRF2) pathway were investigated [134]. siRNA downregulated MALAT1, leading to the suppression of Keap1. NRF2, when dissociated from Keap1, translocates to the nucleus, leading to the transcription of antioxidant and detoxifying genes. Thus, lncRNA MALAT1 modulated antioxidant defense in diabetic retinopathy via its interaction with the Keap1/NRF2 pathway.

### 5.12. lncRNA MEG3

Tong et al. [104] studied the expression of lncRNA MEG3 in the ARPE-19 cell line. It was reported that the upregulation of MEG3 led to the downregulation of miR-34a (the miRNA responsible for the inflammatory response and apoptosis in diabetic retinopathy). The upregulation of miR-34a suppressed SIRT1, which led to NF-κB signaling activation, resulting in inflammation. This is in consonance with our previous study wherein we elucidated the regulatory potential for crosstalk between the NF-κB1 and NRF2 signaling pathways in inflammation [135].

## 6. ncRNAs and Epigenetic Mechanisms of Diabetes

Epigenetic mechanisms, including DNA methylation, the silencing of miRNA and histone modification, can regulate the etiopathogenesis of disease. Indeed, epigenetic events may enhance the expression of inflammatory genes associated with diabetes and its complications [92]. Further, it has been reported that gestational diabetes is associated with DNA methylation and epigenetic alternations to miRNAs [136]. In a study [137] including 252 T1D patients, high levels of CpG-373 and CpG-456 methylation were observed in whole-blood samples, suggesting a relationship between DNA methylation and T1D. Interestingly, Glaich et al. showed a correlation of miRNA expression with CpG methylation in WT R1 mouse embryonic stem cells (ESCs) and DNA methyl transferase triple-knockout embryonic stem cells (DNMT TKO ESCs), indicating that DNA methylation affected the biogenesis of miRNAs [138].

A study [139] investigating the relationship between lncRNA HOTAIR and a histone methyltransferase, polycomb-repressive complex 2 (PRC2), in HRECs was conducted using 3-deazaneplanocin A (DZNep), a histone methylation inhibitor. Glucose-induced increases in PRC2 components such as *SUZ12*, *EED* and *EZH2* transcripts were reduced by DZNep. Significant downregulation of lncRNA HOTAIR, *VEGF-A* and *P300* was also observed following DZNep treatment. *EZH2* was reported to be involved in the transcriptional regulation of lncRNA HOTAIR and several other downstream genes associated with hyperglycemia. High glucose promoted the binding of HOTAIR to *EZH2* and *P300*, which modulated the transcriptional regulation of *VEGF-A*. Stable epigenetic DNA methylation was observed across HOTAIR in hyperglycemic conditions. Further, demethylating agent 5-aza-2′-deoxycytidine (5-aza-dC) was used to investigate [139] the effect of DNA methylation on the expression of HOTAIR. *DNMT3A*, *DNMT3B* and *DNMT1* RNA levels were suppressed, while lncRNA HOTAIR was upregulated following 5-aza-dC administration. Moreover, a glucose-induced increase in *VEGF-A* mRNA was prevented by inhibiting DNA methyltransferases. The epigenetic regulation of inflammation by lncRNA MALAT1 in diabetic retinopathy was studied [140] using human retinal endothelial cells (HRECs) and vitreous humor from diabetic patients. lncRNA MALAT1 inhibits the expression of inflammatory cytokines via PRC2 components associated with it. DZNep downregulated lncRNA MALAT1, TNF-α and PRC2 components. The inhibition of DNA methyltransferase (DNMT1) by 5-aza-dC or zebularine enhanced the expression of lncRNA MALAT1, TNF-α, IL-1β and IL-6.

The interaction of lncRNA Dnm3os with nuclear proteins was studied [92] in human macrophages derived from T2D patients. It was reported that lncRNA Dnm3os interacts with nuclear proteins, such as interleukin enhancer binding factor-2 (ILF-2), as well as nucleolin. Further, it was reported that the macrophage inflammatory phenotype can be regulated by the Dnm3os–nucleolin interaction through epigenetic mechanisms such as chromatin relaxation and enhanced permissive histone modifications.

Interestingly, Witkowski et al. [141] studied the epigenetic regulation of tissue factor by miR-19a using plasma samples of diabetic patients as well as human monocytes (THP-1) and human microvascular endothelial cells (HMEC). The plasma expression of miR-19a was strongly correlated with miR-126. It was reported that miR-19a inhibited the expression of tissue factor at the mRNA and protein levels. Further, miR-126 and miR-19a operated in concert to display anti-thrombotic properties mediated via the regulation of tissue factor expression. This may have important implications in the pathogenesis of diabetes, including diabetes-related thrombogenicity.

The epigenetic modification of miR-200b in diabetic vasculopathy was studied [142] using human microvascular endothelial cells (HMECs). Treatment with 5-aza-dC upregulated miR-200b in hyperglycemic conditions. High glucose caused marked hypomethylation of the miR-200b promoter. High glucose also suppressed DNA methyltransferase 1 (DNMT1) and DNA methyltransferase 3A (DNMT3A). Furthermore, it was reported that such downregulation of DNMT1 and DNMT3A could be inhibited by miR-200b. HMECs supplemented with S-adenosylmethionine (SAM), a methyl-group donor, downregulated miR-200b by correcting promoter methylation, and alleviated endothelial dysfunction. Thus, SAM restored vascular function in diabetic wounds via the downregulation of miR-200b by methylating the miR-200b promoter.

## 7. Clinical Trials on Diabetes-Associated Noncoding RNAs

The NCI Clinical Trials Registry (www.clinicaltrials.gov; accessed on 31 May 2022) reports a large number of studies for diabetes (18,168 results as of May 2022) and related complications. However, clinical trials involving diabetes-associated noncoding RNAs are relatively fewer in number (57 as of May 2022). We have previously discussed the challenges and opportunities in clinical trials in resource-limited settings, which help to understand the relevance of various factors that play crucial roles in ensuring good clinical practice [143]. Noncoding RNAs, including microRNAs and lncRNAs, were evaluated from different biological matrices such as plasma, serum, tissues, cord blood, etc. These noncoding RNAs may serve as potential biomarkers for the prognostication of diabetes. Table 6 summarizes clinical trials on diabetes-associated noncoding RNAs for the better understanding of the reader.

## 8. Functional Relevance of Dysregulated Noncoding RNAs

**Figure 6:**
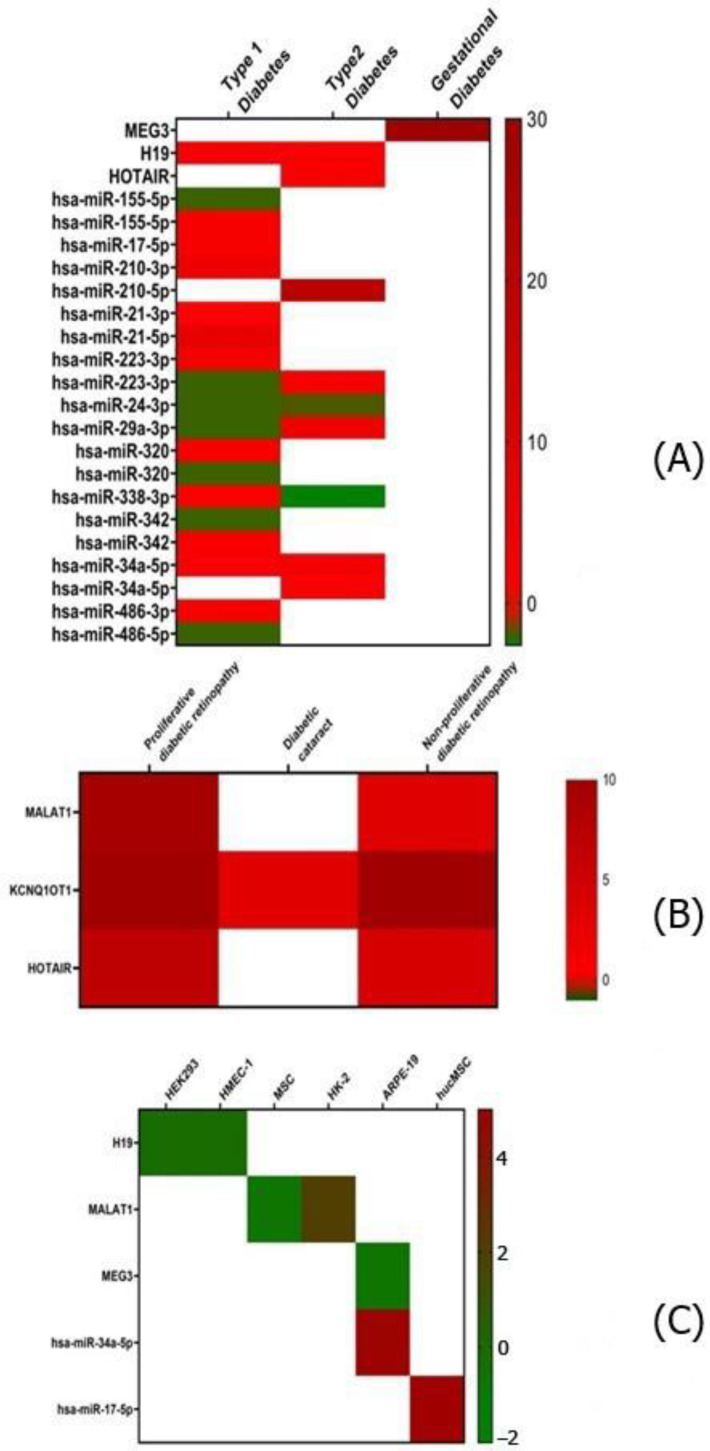
**Heatmaps of common noncoding RNAs as putative markers for diabetes:** Heatmaps of dysregulated noncoding RNAs commonly upregulated (red) and downregulated (green) in diabetes: (**A**) Differential microRNA and lncRNA expression in type 1 diabetes, type 2 diabetes and gestational diabetes. (**B**) Differential lncRNA expression in diabetes-associated complications. (**C**) Differential miRNA and lncRNA expression in cell line studies in diabetes.

Dysregulated noncoding RNAs that are common to two or more studies in the literature and may serve as important putative markers in diabetes and related complications have been enumerated in Table 7. These ncRNAs are involved in the regulation of various metabolic and pathophysiological processes by targeting different genes. Further, we classified these dysregulated ncRNAs based on diabetes type, diabetes-associated complications and in vitro cell line studies and represented them as heatmaps. Figure 6A represents dysregulated lncRNAs and miRNAs involved in different types of diabetes. lncRNA H19, hsa-miR-17-5p, hsa-miR-210-3p, hsa-miR-21-3p, hsa-miR-21-5p, hsa-miR-320, hsa-miR-338-3p, hsa-miR-342, hsa-miR-34a-5p and hsa-miR-486-3p are commonly upregulated in type 1 diabetes, whereas hsa-miR-155-5p, hsa-miR-223-3p, hsa-miR-24-3p, hsa-miR-29a-3p and hsa-miR-486-5p are downregulated. lncRNA H19, HOTAIR, hsa-miR-210-5p, hsa-miR-223-3p, hsa-miR-29a-3p and hsa-miR-34a-5p are commonly upregulated in type 2 diabetes whereas, hsa-miR-24-3p and hsa-miR-338-3p are downregulated. lncRNA MEG3 is uniquely upregulated in gestational diabetes. Figure 6B represents the expression levels of lncRNAs in diabetes-related complications. MALAT1 is upregulated in both proliferative diabetic retinopathy and non-proliferative diabetic retinopathy. KCNQ1OT1 is upregulated in diabetic cataract, proliferative diabetic retinopathy and non-proliferative diabetic retinopathy. HOTAIR is upregulated in both proliferative diabetic retinopathy and non-proliferative diabetic retinopathy. Figure 6C represents upregulated and downregulated lncRNAs and miRNAs in different cell lines. lncRNA H19 is downregulated in the HEK293 and HMEC-1 cell lines; MALAT1, a highly conserved lncRNA, is upregulated in HK2 and downregulated in MSCs and HUVECs; MEG3 is downregulated, whereas hsa-miR-34a-5p is upregulated, in ARPE-19 cells; and hsa-miR17-5p is upregulated in the hucMSC cell line.

**Figure 7:**
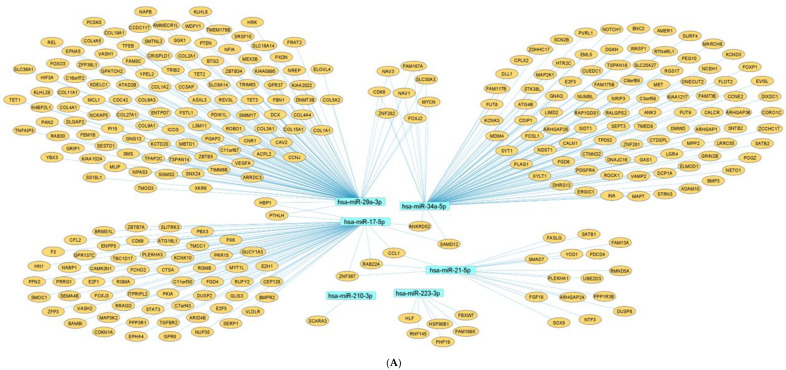

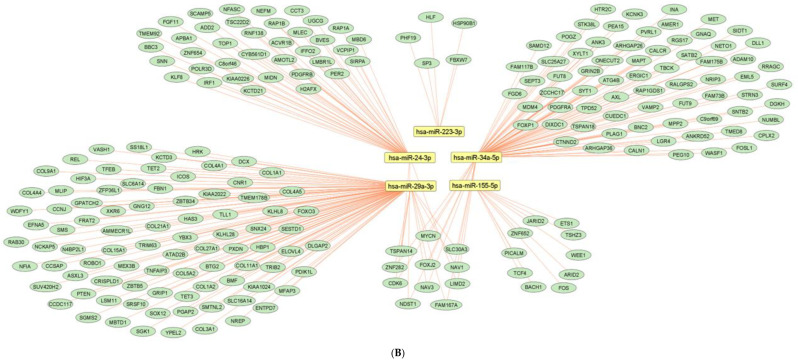

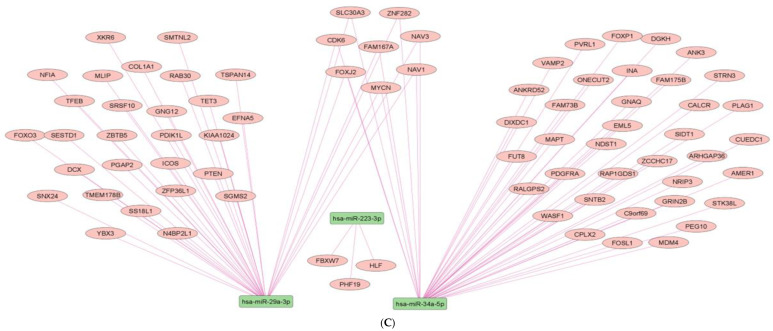
**Common dysregulated miRNA–target networks in diabetes:** (**A**) Human upregulated microRNA–gene target network: Topology of networks of commonly upregulated miRNAs in diabetes showing interaction network of functionally enriched miRNAs with their targets, with 305 nodes and 314 edges. The networks were constructed using Cytoscape 3.9.1 [84]. Using Mienturnet [85], the miRNA–target interactions were identified by TargetScan [86], and the functionally enriched miRNAs were generated using the KEGG database [87]. (**B**) Human downregulated microRNA–gene target network: Topology of networks of commonly downregulated miRNAs in diabetes showing interaction network of functionally enriched miRNAs with their targets, with 225 nodes and 231 edges. The networks were constructed using Cytoscape 3.9.1 [84]. Using Mienturnet [85], the miRNA–target interactions were identified by TargetScan [86], and the functionally enriched miRNAs were generated using the KEGG database [87]. (**C**) Human upregulated and downregulated microRNA–gene target network: Topology of networks of commonly upregulated and downregulated miRNAs in diabetes showing interaction network of functionally enriched miRNAs with their targets, with 79 nodes and 84 edges. The networks were constructed using Cytoscape 3.9.1 [84]. Using Mienturnet [85], the miRNA–target interactions were identified by TargetScan [86], and the functionally enriched miRNAs were generated using the KEGG database [87].

**Figure 8:**
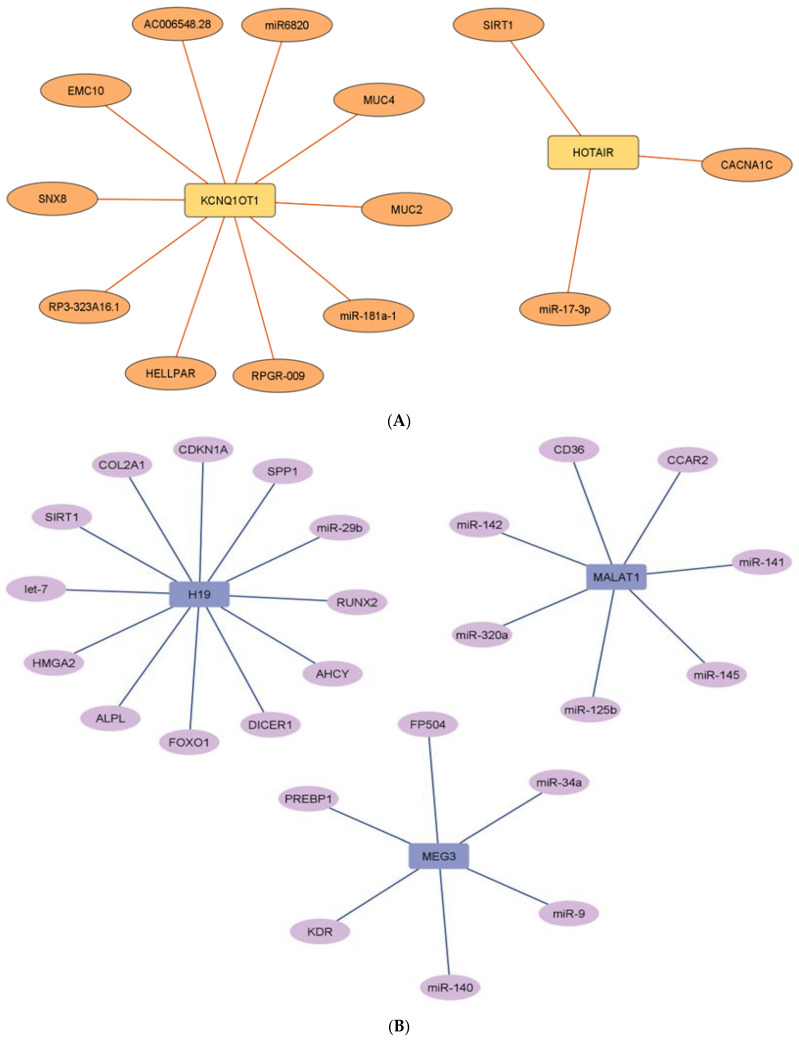

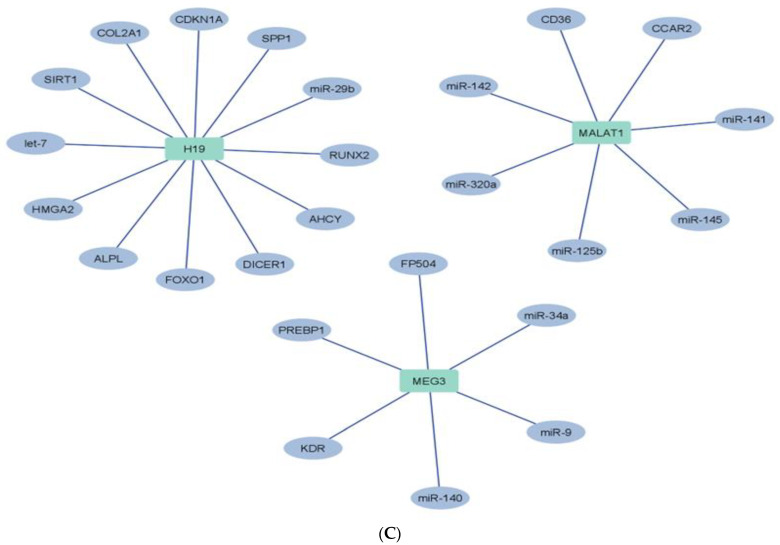
**Common dysregulated lncRNA–target networks in diabetes:** (**A**) Human upregulated lncRNA–gene target network: Topology of networks of commonly upregulated lncRNAs implicated in diabetes in humans showing interaction network of lncRNAs with their targets, with 15 nodes and 13 edges. The networks were constructed using Cytoscape 3.9.1 [84]. The lncRNA–target interactions were identified by lncRNA2target v3.0 [93] and LncRRIsearch [94]. (**B**) Human downregulated lncRNA–gene target network: Topology of networks of commonly downregulated lncRNAs in diabetes showing interaction network of lncRNAs with their targets, with 28 nodes and 25 edges. The networks were constructed using Cytoscape 3.9.1 [84]. The lncRNA–target interactions were identified by lncRNA2target v3.0 [93] and LncRRIsearch [94]. (**C**) Human upregulated and downregulated lncRNA–gene target network: Topology of networks of commonly upregulated and downregulated lncRNAs in diabetes showing interaction network of lncRNAs with their targets, with 28 nodes and 25 edges. The networks were constructed using Cytoscape 3.9.1 [84]. The lncRNA–target interactions were identified by lncRNA2target v3.0 [93] and LncRRIsearch [94].

Biological networks help us to understand the roles and targets of different noncoding RNAs involved in the progression of a disease. The networks are useful in elucidating the mechanism by which ncRNA acts on its target and for prioritizing targets that play key roles in disease pathogenesis. Figure 7A,B represent common upregulated and downregulated miRNA–target networks in diabetes and associated complications. Further, Figure 8A,B represent common upregulated and downregulated lncRNA–target networks in diabetes.

Finally, Table 8 elucidates the functional relevance of dysregulated noncoding RNAs for the benefit of the reader. These commonly modulated noncoding RNAs that appear in multiple studies on diabetes, along with their targets, may represent a subset of putative signature biomarkers that may be exploited for candidate biomarker discovery and the identification of theranostic approaches for more effective management of the disease.

## 9. Conclusions and Future Perspectives

Despite extensive research and advancements in understanding the pathophysiology of diabetes, there exist several limitations in diabetes care. Given its multiple types and uncertain occurrence, diabetes remains an intractable disorder. Robust biomarkers and accurate therapeutic intervention are of prime necessity in tackling the disorder in the most efficient manner. Considering the rapid advancements in biotechnology, research with respect to the identification and isolation of significant prognostic biomarkers for diabetes has undergone a critical paradigm shift. Herein, we delineate miRNA or lncRNA biological networks as well as key miRNA or lncRNA targets in diabetes. It is noted that various ncRNAs are highly dysregulated in diabetes, yielding significant leads in the identification of putative biomarkers for diabetes. The delineation of the ncRNA interactome in diabetes, as well as putative targets, will aid in expediting the identification of novel biomarkers in the discovery pipeline for diabetes. Experimental work tailored towards enhancing our understanding of the ncRNA interactome is necessary to assess these novel biomarkers for therapeutic intervention in the management of diabetes.

## Figures and Tables

**Table 6:** Clinical trials on diabetes-associated noncoding RNAs (adapted from www.clinicaltrials.gov; accessed on 31 May 2022).

Clinical Trial Number	Phase	Status	Number of Participants	Condition	Study Type	Biological Matrix	Intervention	Comments	References
NCT02843139	-	Completed	535	Obesity	Observational/ Cross-sectional	Serum, whole blood	-	Identification of microRNAs (miRNAs) as potential biomarkers to distinguish obese children with diabetes risk in adulthood	[144]
NCT04176276	-	Completed	300	Diabetic kidney disease	Observational/ Retrospective	Urine, serum	Not required	Pathogenesis of renal disorders and related miRNAs that include miR-25, miR-192, miR-194 and miR-204	[145]
NCT01334684	NA	Unknown	100	Type 2 diabetes (T2D)	Interventional	White blood cells (WBCs)	Metformin	Determination of efficacy of metformin by evaluating miRNA profiles in WBCs	[146]
NCT02459106	-	Active, not recruiting	100	T2D	Observational/ Prospective	Muscle, adipose tissue	-	Determination of effect of miRNAs released from fat tissue on skeletal muscle and their contribution to insulin resistance	[147]
NCT03792607	-	Unknown	35	T2D, Cardiovascular disease (CVD)	Observational/ Prospective	Peripheral blood, CD4^+^ and CD8^+^ T-cells	-	Prediction of development of CVD in T2D patients by studying the interactions between miRNAs and DNA methylation	[148]
NCT02383537	-	Unknown	150	Pregnancy and Diabetes	Observational/ Prospective	Placenta, fat tissue	-	Effect of insulin resistance, fat tissue and miRNA during pregnancy on diabetes	[149]
NCT04891887	NA	Completed	163	T2D	Interventional	-	Health education	Determination of risk factors including miRNA and diabetes for mild cognitive impairment	[150]
NCT01042301	NA	Completed	120	Type 1 diabetes mellitus (T1D)	Interventional/ Non-randomized	Blood	Sampling of blood	Profiles of miRNAs determined as humoral and cellular biomarkers along with other biomarkers in T1D	[151]
NCT03472846	IV	Active, not recruiting	80	Post-menopausal osteoporosis	Interventional/ Non-randomized	Serum	Teriparatide and Prolia (denosumab)	Levels of miRNA determined under osteoanabolic treatment and anti-resorptive treatment	[152]
NCT04011228	-	Unknown	224	Prediabetes, Gestational diabetes	Observational/ Prospective	Saliva	Magnetic resonance spectroscopy	Characterizing miRNAs in pre-diabetics and evaluating miRNAs present in heart fat, liver fat and skeletal muscle	[153]
NCT03027726	NA	Completed	84	Obese children with T2D	Interventional/ Randomized	Peripheral mononuclear cells	Psycho-educational program	Profiling miRNA in mononuclear cells and in circulating exosomes to detect diabetes risk in obese children	[154]
NCT03377335	IV	Unknown	186	T2D	Interventional/ Randomized	Serum	Dapagliflozin and metformin	Determination of the effects of dapagliflozin treatment on miRNAs and cardiometabolic risk	[155]
NCT02861781	-	Recruiting	270	Obesity, Insulin resistance, T2D	Observational/ Prospective	Plasma	Sampling of tissue and blood	Comparison and changes in levels of miRNA in plasma, adipose tissue, muscle and liver	[156]
NCT02768935	NA	Unknown	20	Myocardial infarction	Interventional/ Non-randomized	Plasma, platelets	Sampling of blood	Identification of miRNAs responsible for promoting the differentiation of monocytes	[157]
NCT02245633	-	Unknown	70	Diabetic kidney disease	Observational/ Cross-sectional	Plasma	-	Determining the contribution of miRNA expression and levels of vitamin D in DKD treatment	[158]
NCT04392557	IV	Recruiting	36	T2D	Interventional/ Randomized	-	Metformin/alogliptin (oral), metformin/pioglitazone (pill), metformin/alogliptin/pioglitazone (triple therapy)	To compare anti-inflammatory status in atherosclerosis by evaluating inflammatory miRNA levels	[159]
NCT03682445	NA	Completed	60	T2D	Interventional/ Randomized	Blood	Exercise	Effect of exercise on levels of miRNA-143 and other metabolic and physical parameters	[160]
NCT02011100	NA	Completed	28	T2D, CVD, Metabolic diseases	Interventional/ Randomized	-	Carnosine	Effect of carnosine on risk factors of CVD and diabetes; miRNAs related to action of carnosine may provide more effective therapy	[161]
NCT03971955	NA	Recruiting	40	Auto-immune diabetes	Observational/ Cross-sectional	Blood	Mixed meal	Changes in miRNA levels to characterize autoimmune diabetes	[162]
NCT02316522	-	Active, not recruiting	158	Diabetic nephropathy	Observational/ Prospective	Urine, blood	-	Contribution of epigenetics in diabetic nephropathy	[163]
NCT03890822	-	Recruiting	100	Coronary artery disease (CAD)	Observational/ Prospective	Blood	-	Role of inflammatory mediators and circulating miRNAs in diabetic patients along with CAD progression	[164]
NCT04698720	NA	Recruiting	48	Diabetic foot ulcer (DFU)	Interventional/ Randomized	Blood	Hypnosis and muscle relaxation with guided imagery	Effects of hypnosis and muscle-relaxation therapy and evaluation of physiological indicators, including miRNAs (miR-21 and miR-155), for healing DFU	[165]
NCT02138331	II and III	Unknown	20	T1D	Interventional	Cord-blood-derived exosomes	Mesenchymal stem cell (MSC) exosomes	Effect of exosomal content, including pre-miRNA derived from MSC	[166]
NCT03360981	IV	Unknown	150	CVD and diabetes	Interventional/ Randomized	Serum, epicardial fat	Incretins	Evaluation of epicardial fat for prediction of clinical outcomes of CVD-affected patients	[167]
NCT04889053	-	Recruiting	1400	T2D, Vascular calcification (VC), Coronary artery calcification (CAC)	Observational/ Prospective	Serum	-	Change in levels of miR-32 for early diagnosis of VC (prognostic biomarker)	[168]
NCT03323788	-	Unknown	96	Obesity	Observational/ Prospective	Muscle	Exercise	Increased expression of miR-128, miR-378, miR-10a, miR-422a and miR-30 family; decreased expression of miR-532	[169]
NCT02890693	NA	Recruiting	200	Gestational diabetes	Interventional/ Randomized	Cord blood	Psychosocial therapy	To improve mental health and cardio-metabolic health by evaluating variables, including miRNA	[170]
NCT03903757	-	Unknown	100	Obesity and T2D	Observational/ Prospective	RNA sequencing	RNA sequencing	Identifying and comparing miRNA targets in obese and diabetic patients with controls	[171]
NCT03491241	NA	Completed	60	Pre-diabetes and obesity	Interventional/ Randomized	Tissue biopsy	Hypocaloric diet	Effect of sirtuins and inflammatory axis expression; downregulation of miR-27 and miR-195	[172]
NCT05139914	IV	Not yet recruiting	50	Endothelial dysfunction	Interventional/ Randomized	Plasma	Dapagliflozin and placebo	Effect of miRNA and other biomarkers on T2D patients and vascular health	[173]
NCT03264976	-	Not yet recruiting	200	Diabetic retinopathy (DR)	Observational/ Prospective	Serum	Diagnostic test	Role of exosomal miRNAs present in serum	[174]
NCT03546062	-	Completed	30	T2D patients with heart transplant	Observational	Heart tissue	Heart transplant	Evaluation of miRNAs and inflammatory markers associated with cardiac disease progression	[175]
NCT05147961	NA	Not yet recruiting	300	Pre-diabetes	Interventional/ Randomized	Lymphocytes, plasma	Standard care	Prediction of disease risk and determination of prevention measures using miRNAs as biomarkers	[176]
NCT02686177	IV	Completed	50	T2D	Interventional/ Randomized	Progenitor cells	Liraglutide, Metformin or sulfonylurea	Effect of glucagon-like peptide receptor (GLP-1) on angiogenesis and AngiomiR-126 levels compared before and after treatment	[177]
NCT04657367	-	Recruiting	10,000	Pre-diabetes, Diabetes, Dysglycemia, Obesity	Observational/ Prospective	Plasma	-	Plasma miRNAs assessed for diabetes registry	[178]
NCT02407626	NA	Terminated	2	Myocardial ischemia	Interventional/ Randomized	-	Propofol and sevoflurane	Myocardial protection of diabetic patients while undergoing surgery and evaluation of miR-144, miR-125b and miR-208a	[179]
NCT04787952	-	Completed	40	Obesity	Observational/ Prospective	Brown adipose tissue (BAT)	Cold exposure	Role of miRNA in activation of BAT	[180]
NCT02282423	NA	Terminated	26	Obesity and Diabetes	Interventional/ Non-randomized	Muscle tissue	Instructions for diet and exercise	Muscle processed to isolate miRNA and mRNA for study	[181]
NCT03476460	II	Completed	269	Kidney disease	Interventional/ Randomized	-	Sodium chloride (oral and intravenous)	Effect of sodium chloride to prevent contrast nephropathy; miRNAs and other biomarkers evaluated	[182]
NCT04617405	-	Recruiting	300	Pregnancy and diabetes	Observational/ Prospective	Serum, plasma	No interventions	Changes in inflammatory and hormonal factors during pregnancy	[183]
NCT02410005	II and III	Terminated	56	Diabetic nephropathy	Interventional/ Randomized	Urine	Calcitriol and Losartan	Discovery of biomarkers (miRNAs) for kidney disease in T2D	[184]
NCT03804411	IV	Recruiting	800	T2D	Interventional/ Randomized	-	Standard treatment	Determination of genetic markers (miR-21, miR-27, miR-125 and miR-126) for prediction of response to hypoglycemic therapy	[185]
NCT04972890	II and III	Recruiting	12	Erectile dysfunction with DM	Interventional/ Randomized	Mesenchymal stem cells (MSC)	Stem cells	Treating erectile dysfunction using umbilical cord MSC-derived miR-16 and miR-126	[186]
NCT05079399	-	Not yet recruiting	192	Diabetic retinopathy	Observational/ Prospective	Blood	Blood sample	Sequencing of miRNA from angiogenic cells	[187]
NCT04638556	-	Recruiting	300	Peripheral neuropathy (PN)	Observational/ Cross-sectional	Plasma	Diagnostic test	Effect of lncRNAs on PN by regulation of miR-146a	[188]
NCT04924504	-	Recruiting	24	Pregnancy and diabetes	Observational/ Prospective	Serum, plasma	No interventions	Profiling of miRNA isolated from exosomes and other proteins to determine insulin resistance	[189]
NCT05172089	-	Recruiting	420	Diabetic foot ulcer (DFU)	Observational/ Prospective	DFU tissue	-	Determination of disruption of barrier function in the repair of DFU by miRNAs	[190]
NCT04856683	-	Recruiting	1100	Frailty	Observational	Blood	-	Profiling of circulating miRNAs from patients suffering with gonadal and adrenal disease	[191]
NCT02768987	NA	Completed	50	T1D	Interventional	-	Bright bodies (weight-management program)	Effect of physical activities on obese T1D patients	[192]
NCT04767750	-	Completed	101	T2D, Cancer	Observational	Blood	Collection of blood sample	To study lncRNA H19’s role in regulating the expression of insulin-like growth factor 1 receptor (IGF-1R)	[193]

**Table 7:** Noncoding RNAs as putative markers for diabetes.

Sr. no.	ncRNA	Disease	Upregulated /Downregulated	References
1	H19	Diabetes mellitus	Upregulated	[91]
2	H19	Human dermal microvascular endothelial cells (HMEC-1) and human embryonic kidney cells 293 (HEK293)	Downregulated	[110]
3	HOTAIR	Non-proliferative diabetic retinopathy, proliferative diabetic retinopathy	Upregulated	[59]
4	HOTAIR	Type 2 diabetes	Upregulated	[51]
5	hsa-miR-155-5p	Type 1 diabetes	Downregulated	[35]
6	hsa-miR-155-5p	Type 1 diabetes	Upregulated	[33]
7	hsa-miR-17-5p	Human umbilical cord mesenchymal stem cells (hucMSCs)	Upregulated	[99]
8	hsa-miR-17-5p	Type 1 diabetes	Upregulated	[35]
9	hsa-miR-210-3p	Type 1 diabetes	Upregulated	[33]
10	hsa-miR-210-5p	Type 2 diabetes	Upregulated	[49]
11	hsa-miR-21-3p	Type 1 diabetes	Upregulated	[33]
12	hsa-miR-21-5p	Type 1 diabetes	Upregulated	[33]
13	hsa-miR-223-3p	Type 1 diabetes	Downregulated	[35]
14	hsa-miR-223-3p	Type 1 diabetes	Upregulated	[105]
15	hsa-miR-223-3p	Type 2 diabetes	Upregulated	[58]
16	hsa-miR-24-3p	Type 1 diabetes	Downregulated	[35]
17	hsa-miR-24-3p	Type 2 diabetes	Downregulated	[54]
18	hsa-miR-29a-3p	Type 1 diabetes	Downregulated	[35]
19	hsa-miR-29a-3p	Type 2 diabetes	Upregulated	[45]
20	hsa-miR-320	Type 1 diabetes	Upregulated	[33]
21	hsa-miR-320	Type 1 diabetes	Downregulated	[35]
22	hsa-miR-338-3p	Type 1 diabetes	Upregulated	[33]
23	hsa-miR-338-3p	Type 2 diabetes	Downregulated	[56]
24	hsa-miR-342	Type 1 diabetes	Upregulated	[33]
25	hsa-miR-342	Type 1 diabetes	Downregulated	[35]
26	hsa-miR-34a-5p	Human adult retinal pigment epithelial cells (ARPE-19)	Upregulated	[104]
27	hsa-miR-34a-5p	Type 2 diabetes patients, type 1 diabetes	Upregulated	[46]
28	hsa-miR-34a-5p	Type 2 diabetes	Downregulated	[45]
29	hsa-miR-486-3p	Type 1 diabetes	Upregulated	[35]
30	hsa-miR-486-5p	Type 1 diabetes	Downregulated	[35]
31	KCNQ1OT1	Diabetic cataract	Upregulated	[103]
32	KCNQ1OT1	Proliferative diabetic retinopathy, non-proliferative diabetic retinopathy	Upregulated	[116]
33	MALAT1	Diabetic retinopathy	Upregulated	[102]
34	MALAT1	HK-2 (human kidney 2) cells	Upregulated	[113]
35	MALAT1	Human bone marrow-derived mesenchymal stem cells (MSCs)	Downregulated	[98]
36	MALAT1	Non-proliferative diabetic retinopathy, proliferative diabetic retinopathy	Upregulated	[59]
37	MEG3	Human adult retinal pigment epithelial cells (ARPE-19)	Downregulated	[104]
38	MEG3	Gestational diabetes	Upregulated	[41]

**Table 8:** Functional relevance of dysregulated noncoding RNAs.

Noncoding RNAs	Biological Functions	Reference
hsa-miR-155-5p	Negative correlation with glycated hemoglobin (HbA1c) levels	[33]
	Negative correlation with insulin-dose-adjusted HbA1c (IDAA1c) levels	
	Regulation of Fas-associated protein with death domain (FADD)	
	Regulation of (C-X-C motif chemokine ligand 8/interleukin 8) CXCL8/IL8	
	Targets phosphoinositide-3-kinase regulatory subunit 1 (PIK3R1) gene	
hsa-miR-210-3p	Negative correlation with thyroid-stimulating hormone (TSH) levels	[33]
	Negative correlation with insulin-dose-adjusted HbA1c (IDAA1c) levels	
	Targets protein tyrosine phosphatase non-receptor type 1 (PTPN1) gene participating in insulin signaling pathway	
has-miR-210	Linked with hypoxia pathway and upregulated in response to hypoxia-inducible factors (HIFs)	[49]
hsa-miR-29a	Negative correlation with serum sestrin2 levels	[194]
	Suppresses IRS-1, leading to decreased uptake of glucose into the cell	
hsa-miR-17-5p	Downregulates PTEN and activates AKT/HIF-1α/VEGF pathway to promote proliferation, migration and tube formation in HG-treated human umbilical vascular endothelial cells (HUVECs)	[195]
ANRIL GAS5 HOTAIR MALAT1 MEG3	Positive correlation with senescence-related biomarkers (p16, p21, p53 and β-galactosidase) and pro-inflammatory biomarkers (IL1- β, MCP1, IL6 and TNF-α)	[51]
hsa-miR-34a	Decreased SIRT1 activity resulting in senescence of pancreatic β-cells	[47]
hsa-miR-223-3p	Targets HSP90P1 regulating GRP94 expression, which is essential for development of pancreatic β-cells	[196]

## Data Availability

Data sharing not applicable.

## Abbreviations

T1D	Type 1 diabetes
T2D	Type 2 diabetes
GDM	Gestational diabetes mellitus
IGF	Insulin-like growth factors
GAS6	Growth arrest-specific gene 6 protein
VKDP	Vitamin K-dependent protein
ncRNA	Noncoding RNA
miRNA	microRNA
lncRNA	Long noncoding RNA
ceRNA	Competing endogenous RNA
MSC	Mesenchymal stem cells
HK-2	Human Kidney-2 cells
HUVEC	Human umbilical vein endothelial cells
HepG2	Human hepatocellular carcinoma
hRECs	Human retinal endothelial cells
hRMECs	Human retina microvascular endothelial cells
ARPE-19	Adult retinal pigment epithelial cells
PBMCs	Peripheral-blood mononuclear cells
NPDR	Non-proliferative diabetic retinopathy
HMEC-1	Human dermal microvascular endothelial cells
IRS1	Insulin receptor substrate 1
HPA-V	Human preadipocytes-visceral
INSR	Insulin receptor
PDPK1	Phosphoinositide-dependent kinase-1
ER-α	Estrogen receptor-α
PTC	Papillary thyroid cancer
PDE4	Phosphodiesterase 4
PRC2	Polycomb-repressive complex 2
5-aza-dC	5-aza-2′-deoxycytidine
DNMT	DNA methyltransferase
ARPE-19	Human adult retinal pigment epithelial cells
PDR	Proliferative diabetic retinopathy
DKD	Diabetic kidney disease
DR	Diabetic retinopathy
DN	Diabetic nephropathy
